# Peripheral Blood DNA Methylation Signatures and Response to Tofacitinib in Moderate-to-severe Ulcerative Colitis

**DOI:** 10.1093/ecco-jcc/jjad129

**Published:** 2023-08-01

**Authors:** Vincent Joustra, Andrew Y F Li Yim, Sara van Gennep, Ishtu Hageman, Tristan de Waard, Evgeni Levin, Peter Lauffer, Wouter de Jonge, Peter Henneman, Mark Löwenberg, Geert D’Haens

**Affiliations:** Department of Gastroenterology and Hepatology, Amsterdam UMC, University of Amsterdam, Amsterdam, The Netherlands; Amsterdam Gastroenterology Endocrinology Metabolism, Amsterdam UMC, University of Amsterdam, Amsterdam, The Netherlands; Amsterdam Gastroenterology Endocrinology Metabolism, Amsterdam UMC, University of Amsterdam, Amsterdam, The Netherlands; Genome Diagnostics Laboratory, Department of Human Genetics, Amsterdam UMC, University of Amsterdam, Amsterdam, The Netherlands; Amsterdam Reproduction and Development, Amsterdam UMC, University of Amsterdam, Amsterdam, The Netherlands; Tytgat Institute for Liver and Intestinal Research, Amsterdam UMC, University of Amsterdam, Amsterdam, The Netherlands; Department of Gastroenterology and Hepatology, Amsterdam UMC, University of Amsterdam, Amsterdam, The Netherlands; Amsterdam Gastroenterology Endocrinology Metabolism, Amsterdam UMC, University of Amsterdam, Amsterdam, The Netherlands; Department of Gastroenterology and Hepatology, Amsterdam UMC, University of Amsterdam, Amsterdam, The Netherlands; Amsterdam Gastroenterology Endocrinology Metabolism, Amsterdam UMC, University of Amsterdam, Amsterdam, The Netherlands; Tytgat Institute for Liver and Intestinal Research, Amsterdam UMC, University of Amsterdam, Amsterdam, The Netherlands; Horaizon, Bv, Delft, The Netherlands; Horaizon, Bv, Delft, The Netherlands; Amsterdam Gastroenterology Endocrinology Metabolism, Amsterdam UMC, University of Amsterdam, Amsterdam, The Netherlands; Department of Pediatric Endocrinology, Emma Children’s Hospital, Amsterdam University Medical Center, University of Amsterdam, Amsterdam, The Netherlands; Amsterdam Gastroenterology Endocrinology Metabolism, Amsterdam UMC, University of Amsterdam, Amsterdam, The Netherlands; Tytgat Institute for Liver and Intestinal Research, Amsterdam UMC, University of Amsterdam, Amsterdam, The Netherlands; Department of Surgery, University of Bonn, Bonn, Germany; Amsterdam Gastroenterology Endocrinology Metabolism, Amsterdam UMC, University of Amsterdam, Amsterdam, The Netherlands; Genome Diagnostics Laboratory, Department of Human Genetics, Amsterdam UMC, University of Amsterdam, Amsterdam, The Netherlands; Amsterdam Reproduction and Development, Amsterdam UMC, University of Amsterdam, Amsterdam, The Netherlands; Department of Gastroenterology and Hepatology, Amsterdam UMC, University of Amsterdam, Amsterdam, The Netherlands; Amsterdam Gastroenterology Endocrinology Metabolism, Amsterdam UMC, University of Amsterdam, Amsterdam, The Netherlands; Department of Gastroenterology and Hepatology, Amsterdam UMC, University of Amsterdam, Amsterdam, The Netherlands; Amsterdam Gastroenterology Endocrinology Metabolism, Amsterdam UMC, University of Amsterdam, Amsterdam, The Netherlands

**Keywords:** Epigenetics, biomarkers, personalised medicine

## Abstract

**Introduction:**

Predictive biomarkers for treatment efficacy of ulcerative colitis [UC] treatments are lacking. Here, we performed a longitudinal study investigating the association and potential predictive power of genome-wide peripheral blood [PB] DNA methylation signatures and response to tofacitinib treatment in UC.

**Methods:**

We recruited moderate-to-severe UC patients starting tofacitinib treatment, and measured PB DNA methylation profiles at baseline [T1], after 8 weeks [T2], and in a subset [*n* = 8] after a median of 20 weeks [T3] using the Illumina Infinium HumanMethylation EPIC BeadChip. After 8 weeks, we distinguished responders [R] from non-responders [NR] based on a centrally read endoscopic response [decrease in endoscopic Mayo score ≥1 or Ulcerative Colitis Endoscopic Index of Severity ≥2] combined with corticosteroid-free clinical and/or biochemical response. T1 PB samples were used for biomarker identification, and T2 and publicly available intraclass correlation [ICC] data were used for stability analyses. RNA-sequencing was performed to understand the downstream effects of the predictor CpG loci.

**Results:**

In total, 16 R and 15 NR patients, with a median disease duration of 7 [4–12] years and overall comparable patient characteristics at baseline, were analysed. We identified a panel of 53 differentially methylated positions [DMPs] associated with response to tofacitinib [AUROC 0.74]. Most DMPs [77%] demonstrated both short- and long-term hyperstability [ICC ≥0.90], irrespective of inflammatory status. Gene expression analysis showed lower FGFR2 [*p*_BH_ = 0.011] and LRPAP1 [*p*_BH_ = 0.020], and higher OR2L13 [p_BH_ = 0.016] expression at T1 in R compared with NR.

**Conclusion:**

Our observations demonstrate the utility of genome-wide PB DNA methylation signatures to predict response to tofacitinib.

## Graphical Abstract

**Graphical Abstract:**
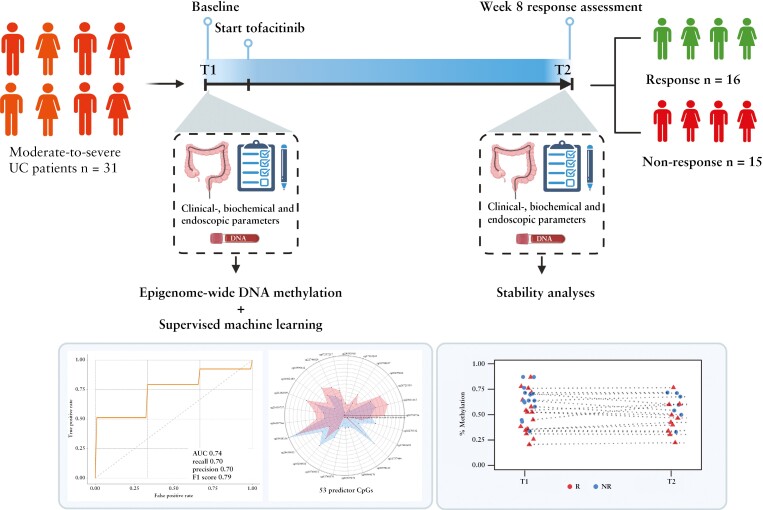

## 1. Introduction

Ulcerative colitis [UC] is a chronic inflammatory condition characterised by continuous mucosal inflammation of the rectum and, to a variable extent, into the colon, and a relapsing and remitting course.^1^ Current biologic treatments registered for UC include therapeutic antibodies directed against tumour necrosis factor [TNF], α4β7 integrin, and the p40 subunit of interleukin-12 and interleukin-23.^2^ Orally available Janus kinase [JAK] inhibitors represent a novel therapy for the treatment of UC patients. These small molecules inhibit the JAK-STAT pathway by blocking one or more JAK receptors. As the first JAK inhibitor approved for UC,^3^ tofacitinib preferentially blocks phosphorylation of JAK1 and JAK3, and to a lesser extent JAK2, leading to reduced dimerisation of STAT1, STAT3, STAT5, and STAT6, which bind to DNA-regulatory elements altering transcription in myeloid and lymphoid cells.^4,5^ As a result, tofacitinib inhibits the effects of γc-containing cytokines [IL-2, IL-4, IL-7, IL-9, IL-15, and IL-21], IFN-y, IL-6, and to a lesser extent IL-12 and IL-23.^5^ Cross-talk between the JAK-STAT signalling cascade and phosphatidylinositol 3-kinase [PI3K], mammalian target of rapamycin [mTOR], protein kinase B [Akt], mitogen-activated protein kinase [MAPK], and extracellular signal-regulated kinase [ERK] pathways have been shown.^6–9^ Moreover, JAK inhibitors have been reported to inhibit the JAK/STAT as well as the PI3K/Akt/mTOR pathway.^10,11^

The phase 3 tofacitinib induction and maintenance trials have shown significant improvements in achieving clinical and endoscopic endpoints compared with placebo.^12^ Yet despite these results, a significant proportion of patients fail to reach mucosal healing [69%] or clinical remission [46%] at Week 8, highlighting the unmet need to develop predictive biomarkers for treatment response to tofacitinib.^12,13^

An increasing body of evidence suggests that epigenetic alterations, such as aberrant DNA methylation, are involved in the pathogenesis of inflammatory bowel disease [IBD].^14–19^ DNA methylation involves the binding of a methyl group to a cytosine–phosphate–guanosine [CpG] dinucleotide, and its presence is often inversely correlated with gene expression when occurring near the transcription start sites.^20^ Mapping aberrant DNA methylation in complex multifactorial diseases has gained increasing interest due to its capability of dynamically responding to environmental factors, thought to act as key triggers of the dysregulated immune response in UC.^21,22^ Previous literature, focused on UC patients, has shown differentially methylated loci across different cell and tissue types to be associated with differences in disease phenotype.^23–26^

Several studies aiming to predict treatment success of tofacitinib, using the transcriptome and proteome, have been conducted in rheumatoid arthritis, psoriasis, and UC patients.^27–29^ However, no study has explored a possible association of the peripheral blood [PB] DNA methylome with response to tofacitinib treatment in UC. In addition, DNA methylation profiles are thought to be superior to RNA and most proteins in providing stable molecular information, highlighting their potential to be used as theranostic biomarkers for UC.^30^

Therefore, this study aimed to explore the association and predictive capabilities of the PB DNA methylome with response to tofacitinib in UC. In addition, we explored the functionality of our identified CpG signals by means of targeted gene expression. Finally, we investigated both short- and long-term stability of the identified predictor CpGs, by longitudinal analyses in a subset of patients at Week 8 and after a median treatment duration of 20 weeks, as well as using publicly available IBD-associated PB DNA methylation long-term stability data.^31^

## 2. Materials and Methods

### 2.1. Patient selection

Prospectively included were 31 adult [aged ≥18 years] patients with moderate-to-severe active UC, defined by a total Mayo score ≥6 points and an endoscopic Mayo subscore of ≥2. Patients were previously diagnosed to have UC in accordance with the ECCO guideline.^32^ All included patients received open-label treatment with 10 mg tofacitinib twice daily. Prior to starting tofacitinib and 8 weeks into treatment, endoscopies [sigmoidoscopies] were performed using the endoscopic Mayo score [EMS] and Ulcerative Colitis Endoscopic Index of Severity [UCEIS] endoscopic activity indices to assess endoscopic response. All endoscopies were videotaped and reviewed through central reading by a single expert endoscopist [GD] blinded for treatment outcome. In addition, clinical (Simple Clinical Colitis Activity Index [SCCAI] and total Mayo score) as well as biochemical (serum C-reactive protein [CRP] and faecal calprotectin) parameters were collected prior to start and after 8 weeks of treatment. Response to tofacitinib was based on a strict composite endpoint, consisting of endoscopic response [decrease in EMS ≥1 or UCEIS ≥2] combined with clinical and/or biochemical response at Week 8 [Table 1]. Following this 8 -week induction period, patients entered regular care follow-up with evaluation of sustained response up to Week 104, using a physician’s global assessment. This study was approved by the medical ethics committee of the Amsterdam University Medical Hospitals [location Academic Medical Hospital] and written informed consent was obtained from all subjects prior to sampling [METC NL57944.018.16 and NL53989.018.15].

**Table 1.** Response criteria

	Description	*N* [%]
Endoscopic response	Decreased endoscopic Mayo score ≥1 or UCEIS score ≥2 compared with baseline^33–35^	16 [51.6]
Biochemical response	≥50% decreased in CRP and faecal calprotectin or CRP ≤5.0 mg/L and faecal calprotectin ≤250 ug/g	18 [58.1]
Clinical response	Decreased total Mayo score ≥3 and ≥30% and decreased rectal bleeding subscore ≥1 or absolute subscore ≤1, or 50 % drop in SSCAI score^36,37^	22 [71]

UCEIS, Ulcerative Colitis Endoscopic Index of Severity; SSCAI, Simple Clinical Colitis Activity Index.

### 2.2. Sample collection and storage

For DNA methylation analyses, PB samples were collected in 4.5-mL EDTA tubes to prevent coagulation. Samples were immediately stored at –80 ºC until further handling. For targeted gene expression analyses, PB samples were collected in 2.0-mL PAXgene Blood RNA tubes and were frozen at –20 ºC for 24 h before storing at –80 ºC until further handling.

### 2.3. DNA isolation, quality control, and pre-processing

Genomic DNA [gDNA] was isolated using the QIAsymphony for consistency, after which the quantity of obtained DNA was assessed using the FLUOstar OMEGA. High-molecular weight DNA was assessed on a 0.8% agarose gel. Next, we batch-randomised high-quality DNA to mitigate batch effects. Subsequently, the gDNA was bisulphite converted using the Zymo EZ DNA Methylation kit, after which whole-genome peripheral blood DNA methylation profiles were quantified using the Illumina Methylation EPIC BeadChip, at the Core Facility Genomics, Amsterdam UMC, Amsterdam, The Netherlands. Using the Bioconductor *minfi*^38,39^ package [version 1.44], the raw methylation data were imported into the R statistical environment [v4.2], after which the raw signals were normalised using functional normalisation.^40^ Next, to increase the probability of identifying true methylation signals rather than genetic variants, probes binding both annotated and unannotated genetic variants [min_maf = 0.01] were removed using Gaphunter^41^ [set to 0.1].

### 2.4. DNA methylation data analyses

**Figure 1.**
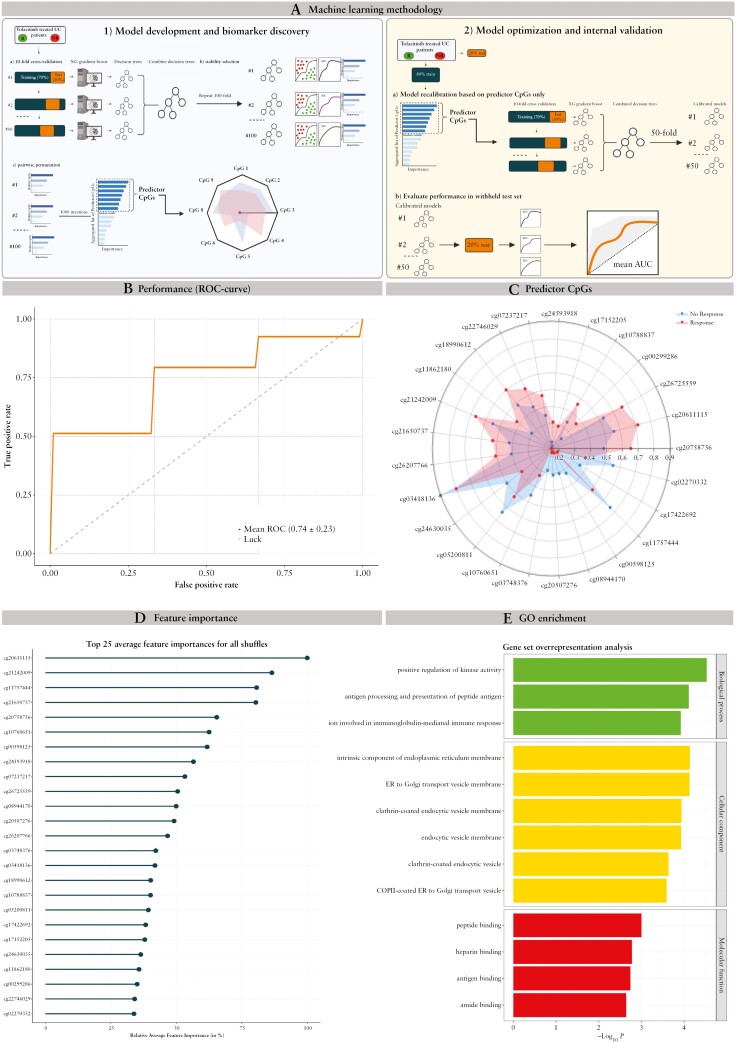
Predictive model using stability selected gradient boosting for response to tofacitinib. A] Machine learning setup and model generation to predict response to tofacitinib. B] Receiver operating characteristics plot showing mean area under the curve [AUC] performance upon internal validation on 20% withheld test set. C] Radar plot presenting the difference in methylation between response [red] and non-response [blue] for the top 25 predictor CpG loci. D] Variable feature importance of the top 25 predictor CpG loci. E] Functional overrepresentation using GO-term analysis of genes annotated to the 53 predictor CpG loci.

For the purpose of epigenetic biomarker discovery, we used a rigorous, supervised machine learning setup focusing on feature selection on samples taken prior to the start of tofacitinib treatment [Figure 1A]. In short, the full cohort of patients was divided into a 70% train and 30% test set, where we used stability selected gradient boosting on the train set. Stability selected gradient boosting is a tree-based, classification technique that uses a stepwise feature selection approach building on existing weaker trees in an effort to minimise the overall prediction error against the observed data^42,43^. To mitigate overfitting, which is a common issue in high-dimensional datasets, we employed 10-fold cross-validation [Step 1a].^44^

In addition, to secure the identification of reliable and robust biomarker signals, we repeated Step 1a a total of 100 times by rearranging the data and applying a different split in 70% train and 30% test set, a step we call ‘stability selection’.^45^ As a result, we generated a 100, 10-fold, cross-validated, decision trees, each with their own ranked list of CpG markers [Step 1b]. We then combined these 100 decision trees and selected the most relevant markers through permutation analysis.^46^ This test ran the model 1000 times, each time randomly removing a CpG and permuting a random variable, evaluating the impact of the permutation on model performance. The CpG loci were ranked based on their relative importance in the marker panel, and only those CpG loci ranked above the imputed random variable were considered ‘predictor CpGs’ and retained for further analysis [Step 1c].

Following this selection of predictor CpG loci, we optimised the predictive model by repeating Step 1a–c on 80% of the original dataset, but this time focused on only the predictor CpG loci, a step we call ‘model recalibration’ [Step 2a]. This resulted in 50 calibrated models which were then evaluated on the 20% withheld test set to distinguish R from NR. The final receiver operating characteristic [ROC] curves were generated from the mean performance of all 50 models [Step 2b]. We used Python version 3.10 [www.python.org], with packages Numpy, Scipy, and Scikits-learn for implementing the model and R version 4.2 [R Foundation, Vienna, Austria] for visualisations.

Post-hoc linear regressions against covariates SCCAI and corticosteroid use were conducted using the Bioconductor *limma* package [v3.56.0] where we regressed the methylation status of each of the predictor CpG loci against SCCAI as a continuous variable and corticosteroid use as a binary variable. Resulting *p*-values were subsequently corrected for multiple testing using the Benjamini–Hochberg method, where predictor CpGs were found to be significantly associated with covariates if their adjusted *p*-values fell below 0.05.

### 2.5. Gene ontology over-representation analysis

Gene ontology [GO]^47,48^ over-representation analysis was performed with gene sets downloaded from MSigDB [https://www.gsea-msigdb.org/gsea/msigdb/index.jsp], using the over-representation test procedures of clusterProfiler [4.6.0].^49^ Gene sets with a false-discovery rate [FDR]-adjusted *p*-value <0.05 were considered significant. Redundancy of over-represented GO terms was removed with the simplify function implemented in clusterProfiler.

### 2.6. Quantification of RNA expression and data processing

Transcriptomic analyses were performed through RNA sequencing. To do so, mRNA was extracted using the QIAsymphony, converted into cDNA, and thereafter sequenced in a 150-bp, paired-ended fashion on the Illumina NovaSeq6000 to a depth of 40 million reads at the Amsterdam UMC Core Facility Genomics. Next, quality control of the reads was done with FastQC [v0.11.8] and summarisation through MultiQC [v1.0]. Raw reads were aligned to the human genome [GRCh38] using STAR [v2.7.0] and annotated using the Ensembl v95 annotation. Post-alignment processing was performed through SAMtools [v1.9], after which reads were counted using the featureCounts function found in the Subread package [v1.6.3].

Differential expression [DE] analysis was performed using the Bioconductor [v3.14] package DESeq2 [v1.38.3] [30] in the R statistical environment [v4.2], where we extracted the expression of the genes that associated with the predictor CpG loci. Differentially expressed genes [DEGs] were defined as genes whose difference presented a Benjamini–Hochberg-adjusted *p*-value <0.05. Visualisations were created in ggplot2^50^ [v3.4.0].

### 2.7. Statistical analysis of clinical data

Descriptive statistics were used to analyse baseline characteristics of all included patients. Continuous data are expressed as median [IQR], and categorical data as frequencies and percentages. Differences in distribution between responders and non-responders were assessed using a chi square test [categorical variables] or Mann–Whitney U test [continuous variables]. Two-tailed probabilities were used with a *p*-value of ≤0.05 considered as statistically significant. Analyses of clinical data were performed in IBM SPSS statistics version 26.

### 2.8. Data availability

Raw DNA methylation data have been made publicly available in the European Genome-phenome Archive [EGA] repository under accession identifier: EGAS00001006968. Raw gene expression data are available upon request to the corresponding author. The code used for the initial cleanup and analyses can be found at [https://gitlab.com/ND91/ucpbtofresp].

## 3. Results

Genome-wide PB DNA methylation signatures were assessed prior to the initiation of tofacitinib treatment in 31 moderate-to-severe UC patients at baseline [T1] and in a subset of patients at Week 8 [T2, *n* = 17] as well as after a median treatment duration of 20 weeks [T3, *n* = 8]. A strict composite endpoint was used, consisting of endoscopic response combined with clinical and/or biochemical response, resulting in 16 R and 15 NR after 8 weeks of tofacitinib treatment. Overall endoscopic response was seen in 16 out of 31 [51.6%], and biochemical and clinical response in 18 [58.1%] and 22 [71%] out of 31 patients at Week 8, respectively [Table 1]. During regular care follow-up until 2 years following the start of tofacitinib, 12 out of 15 [80%] NR at Week 8 discontinued treatment, of whom 11 switched to a different mode of action and one underwent colectomy due to refractory disease. The remaining three NR patients continued on the drug at a dosage of 10 mg twice daily and eventually responded, of whom one achieved endoscopic remission [EMS 0] within 52 weeks and the other two responded [decrease in EMS ≥1] within 2 years. All three patients are currently still on tofacitinib at a dosage of 5 mg twice daily. For the responding patients after 8 weeks of treatment, 14 out of 16 [88%] sustained clear clinical, biochemical, and endoscopic response during 2 years of follow-up. For the remaining two patients, one stopped due to non-compliance and the other was lost to follow-up after 4 months, at which time the patient was in clinical and biochemical remission. None of the responding patients underwent colectomy within 2 years following the start of tofactinib.

At baseline, no significant differences in age [*p* = 0.32], sex [*p* = 0.35], and smoking behaviour [*p* = 0.09] were observed between R and NR. Of 31 patients, 21 [67.7%] were previously exposed to immunomodulators [azathioprine, mercaptopurine, thioguanine, or methotrexate], 22 patients [70.9%] to prior anti-TNF treatment [infliximab, adalimumab, or golimumab], 14 patients [45.2%] to vedolizumab, and three [9.7%] to ustekinumab. The majority of patients had extensive disease [ie, Montreal classification: E3] at baseline endoscopy, and approximately half of the patients needed oral corticosteroids at initiation of tofacitinib treatment, which did not differ between responders and non-responders [*p* = 0.47]. Non-responders showed a significant higher baseline SCCAI score compared with responders [*p* = 0.01]; however, no significant differences in baseline CRP [*p* = 0.07], faecal calprotectin [*p* = 0.36], or endoscopic Mayo score [*p* = 0.74] were observed. Additional baseline characteristics of R and NR are shown in Table 2.

**Table 2.** baseline characteristics

	Responders [n = 16]	Non-responders [n = 15]	p-value
Female, *n* [%]	8 [50]	10 [66.7]	0.35
Age, years, median [IQR]	43.5 [30.5 to 67]	50 [29 to 54]	0.32
Ethnic background, *n* [%] - Caucasian	13 [81.3]	13 [86.7]	0.25
C-reactive protein, mg/L, median [IQR]	3.5 [1.6 to 10.6]	16.6 [4.8 to 27.3]	0.07
Albumin, g/L, median [IQR]	41 [39 to 43.3]	40 [37.5 to 43]	0.56
Faecal calprotectin, ug/g, median [IQR]	2838 [507 to 5309]	1418 [250 to 3387]	0.36
Baseline SCCAI score, median [IQR]	8.5 [6.3 to 10.8]	12 [9 to 13]	**0.01**
Baseline endoscopic Mayo score, *n* [%]			0.74
- Mayo 2	4 [25]	3 [20]	
- Mayo 3	12 [75]	12 [80]	
Disease location, *n* [%]			0.22
- Ulcerative proctitis [E1]	2 [12.5]	-	
- Left-sided UC, distal to splenic flexure [E2]	7 [43.8]	6 [40]	
- Extensive UC, proximal to splenic flexure [E3]	7 [43.8]	9 [60]	
Previous IBD-related surgery, n [%]			
- Appendectomy	1 [6.3]	2 [13.3]	0.51
Previous treatment, n [%]			
- 5-ASA	15 [93.8]	12 [80]	0.25
- Immunomodulator [AZA, 6MP, 6TG, MTX]	9 [64.3]	12 [80]	0.34
- Anti-TNF [IFX, ADA, GOL]	11 [78.6]	11 [73.3]	0.74
- Vedolizumab	10 [62.5]	4 [26.7]	0.05
- Ustekinumab	1 [6.3]	2 [13.3]	0.50
Concomitant medication			
- Prednisone	8 [50]	9 [60]	0.47
Smoking, *n* [%]			0.09
- Active	-	1 [6.7]	
- Ex-smoker	11 [68.8]	5 [33.3]	
- Never smoked	5 [31.3]	9 [60]	

*P-*values in bold are significant.

Abbreviations: 6MP: mercaptopurine; 6TG: thioguanine; ADA: adalimumab; AZA: azathioprine; CpG: cytosine-phosphate-guanine; DMP: differentially methylated position; ECCO: European crohn’s and colitis organization; GOL: golimumab; IBD: inflammatory bowel disease; IFX: infliximab; IQR: interquartile range; MTX: methotrexate; NR: non-responder; PB: peripheral blood; R: responder; SCCAI: simple clinical colitis activity index; UC: ulcerative colitis; UCEIS: Ulcerative Colitis Endoscopic Index of Severity.

Since we were interested in CpGs with predictive potential, we performed stability selected gradient boosting analyses using blood samples that were taken before start of tofacitinib treatment. The best predictive model was able to distinguish R from NR at Week 8 with good accuracy (AUROC [area under ROC] = 0.74, precision = 0.70, recall = 0.70, and F1 score = 0.79) based on a combination of 53 predictor CpGs [Figure 1B]. In addition, our model presented a similar performance for sustained response up to Week 104 [AUC 0.74, precision 0.81, recall 0.56, F1 score 0.63], shown in Supplementary Figure S1. Comparing R with NR indicated that 26 and 27 predictor CpGs presented hyper- and hypo-methylation, respectively [SupplementaryFigure S2 and Supplementary Table S1].

Focusing on the top 25 markers, 15 CpG loci annotated to 14 unique genes, of which six [*MAGI3*, *ERICH1*, *HLA*-*DQB1*, *PALD1*, *BTNL9*, and *LMLN2*] presented hyper-methylation, whereas nine [RPTOR, *CLCA1*, *HLA-DRB1*, *HLA-DRB5*, *OR2L13*, *RP11-526l2.5*, *SPATC1L*, and *MRPL28*] presented hypo-methylation in R relative to NR [Figure 1C and D].

Next, we aimed to explore the functional relevance of our identified DMPs by means of gene ontology [GO] overrepresentation analysis, which yielded significant hits [FDR <0.05] for positive regulation of kinase activity [GO:0033674], antigen processing and presentation of peptide antigen [GO:0048002], and immunoglobulin production involved in immunoglobulin-mediated immune response [GO:0002381] [Figure 1E and Supplementary Table S2]. In short, the genes annotated to the response-associated methylation signature corroborate the mode of action of tofacitinib.

**Figure 2.**
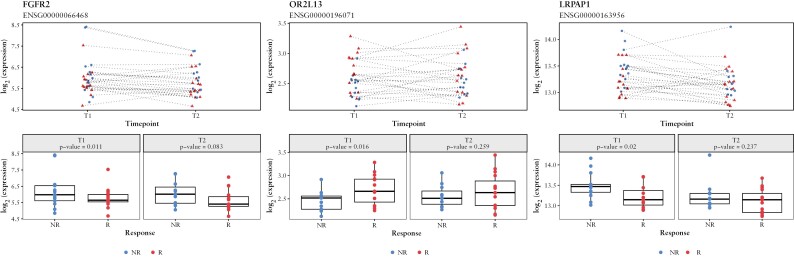
Expression of *FGFR2*, *OR2L13*, and *LRPAP1*. Visualisation of the level of expression (log_2_[counts+1]) for each individual sample before [T1] and at 8 weeks into tofacitinib treatment [T2] time coloured by response. Red represents responders and blue represents non-responders. The top plot represents a scatterplot of the patient over time, with dashed lines connecting samples obtained from the same patient. The bottom plots represent boxplots for T1 and T2 separately, comparing responders and non-responders annotated with the differential expression *p*-value.

In addition, gene expression levels of genes annotated to the 53 predictor CpGs were assessed. Doing so, we observed a significant lower expression of *FGFR2* [*p*_BH_ = 0.011] and *LRPAP1* [*p*_BH_ = 0.020], and a significant higher expression of *OR2L13* [*p*_BH_ = 0.016] at T1, in R compared with NR [Figure 2]. The expression levels of all other genes were not significantly different at T1 and T2 between responders and non-responders [Supplementary Figure S3].

**Figure 3.**
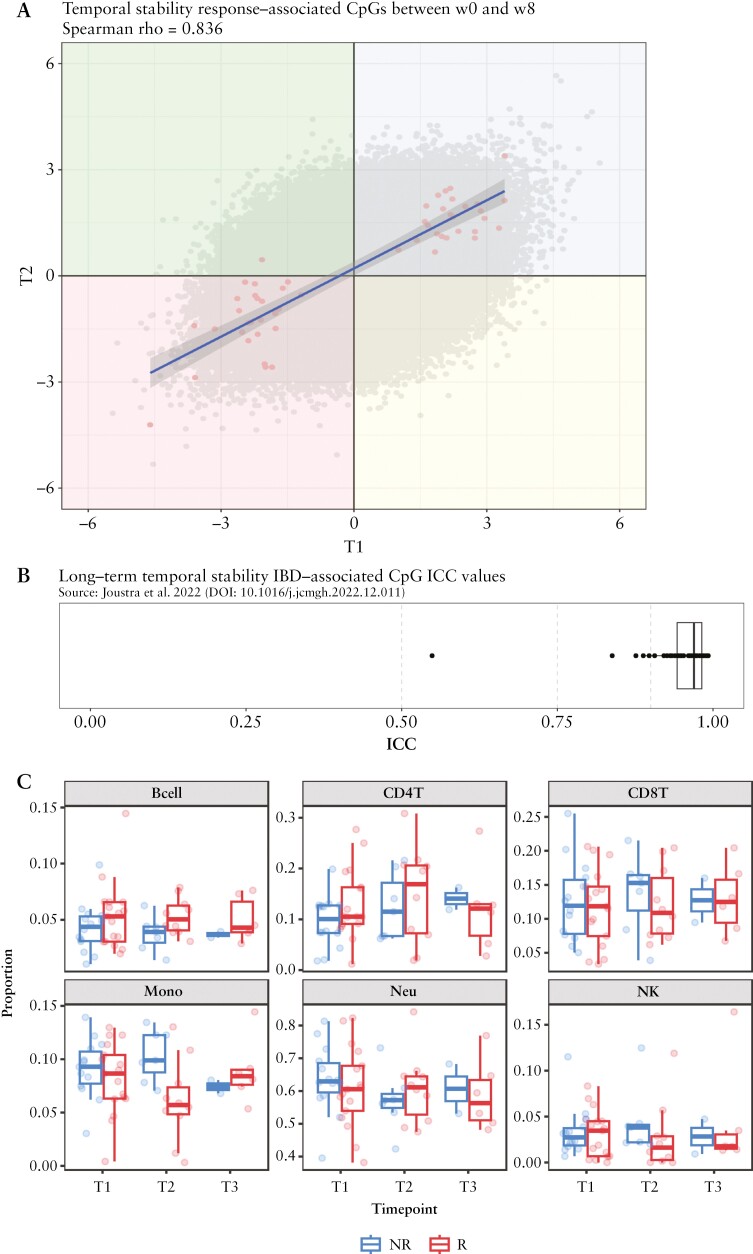
Stability analyses and estimated cell proportion. A] Spearman correlation plot showing the correlation of differential DNA methylation between responders [R] and non-responders [NR] over time. Dots in grey represent CpG loci located on the Illumina EPIC array, dots in red represent the identified predictor CpG loci. The four quadrants represent the associated differential methylation across time. CpGs in the blue quadrant are hypermethylated in both T1 and T2, whereas CpGs in the red quadrant are both hypomethylated in T1 and T2, indicating time stability. CpGs in the green quadrant are, whereas CpGs in the yellow quadrant are, indicating time instability. B] Intraclass correlation coefficients of the 53 predictor CpGs obtained from previous long-term stability analyses.^51^ Grey dashes represent the classification boundaries introduced by Koo and Li,^52^ with blocks representing poor [ICC <0.5], moderate [0.5 ≤ ICC < 0.75], good [0.75 ≤ ICC < 0.9], and excellent [0.9 ≥ICC]. C] Estimated blood cell distribution of the monocytes, NK cells, CD8+ T cells, B cells, CD4+ T cells, and neutrophils using Houseman. The x-axis of each box indicates the difference between responders [R] [red] vs non-responders [NR] [blue]. The y-axis of each box shows the proportion of that particular cell type. A significantly lower proportion of monocytes at T2 were observed for patients responding to tofacitinib.

As temporal stability is a key aspect of robust biomarker development, we next explored the stability of our identified predictor CpGs by correlating the difference in methylation of each CpG at T1 with the difference in methylation of each CpG at T2, after 8 weeks of treatment. High correlation of CpG methylation was detected between T1 and T2 [Spearman rho = 0.83]. Notably [Figure 3A], the 53 predictor CpGs [in black] are either located in the upper right or lower left quadrant, indicating that these CpG loci are consistently differentially methylated at T1 and T2. Further focusing on the predictor CpG loci only, we did not observe significant differential methylation between T1, T2, and T3. Moreover, intraclass correlation [ICC] analysis indicated that 47 [89%] presented good stability [0.75 ≤ ICC < 0.90] of which 34 [64%] presented excellent stability [ICC ≥0.90] between the two or three time points sampled [Figure 3B and Supplementary Figure S4]. Interrogating the long-term stability of the predictor CpGs in a longitudinal cohort of IBD patients corroborated our stability observations, showing that 41 [77%] and three [6%] of the 53 predictor CpGs demonstrated excellent [ICC ≥0.90] and good [ICC 0.75–0.89] long-term stability, respectively [Figure 3B].^51^

Whereas these analyses demonstrate stability of the identified predictor CpGs, implicating their independence of inflammatory status during both induction and maintenance treatment, it is well known that DNA methylation is highly tissue- and cell-type specific.^53,54^ Therefore, the differences in peripheral blood DNA methylation between R and NR at T1 could potentially emerge, as a consequence of differences in cellular composition. To address this issue, we estimated the proportions of six major peripheral blood cell types [CD8+ T cells, CD4+ T cells, natural killer cells, B cells, monocytes, and neutrophils] per sample at each of the three time points, using the method described by Houseman *et al*.^55^ Expectedly, we observed a significant lower proportion of monocytes [*p*_BH_ = 0.02] in R compared with NR at T2.^56,57^ All other cell types were not significantly different between T1, T2, and T3 for R and NR, suggesting that response to tofacitinib is not associated with a difference in cellular composition of the aforementioned cell types at T1 [Figure 3C, Supplementary Table S3]. In addition, the response-associated DMPs did not significantly associate with baseline SCCAI or corticosteroid use [Supplementary Tables S4 and S5].

## 4. Discussion

This is the first study that prospectively explored the association of PB DNA methylation signatures and response to tofacitinib in UC. Using stability selected gradient boosting, we identified a panel of 53 CpG loci that were capable of predicting early combined clinical, biochemical, and endoscopic response with good accuracy [AUROC = 0.74, precision = 0.70, recall = 0.70, and F1 score of 0.79] upon internal validation. Moreover, we observed a similar performance up to 2 years following the start of tofacitinib, using a global physician assessment [AUROC 0.74]. The model’s recall and precision performances of 0.70 indicate that it can identify 70% of true responding patients, potentially resulting in 30% unnecessary treatment with tofacitinib based on the combination of these 53 CpG loci. The F1 score of 0.79 indicates a favourable balance between precision and recall. In addition, we calculated a likelihood ratio of 2.33 with a post-test likelihood of 72.4%, based on previously reported segmental endoscopic response rates.^58^ Since the current trial-and-error approach in treatment selection leads to a significant proportion of patients without sustained response, an increase of 72.4% in selecting true responding patients would have a significant impact on UC treatment. In addition, the majority of our identified predictor CpGs presented extremely high levels of stability through longitudinal analyses, suggesting time-invariant, response-associated, differential methylation.

Several of the identified predictor CpG loci may be linked to the JAK-STAT signalling pathway and could be interesting targets for future studies exploring the biological mechanism behind tofacitinib treatment response. The hypomethylated predictor CpGs cg08944170, cg20507276, and cg03748376 all occurred in the first exon of *OR2L13*, a G-protein coupled olfactory receptor detectable in several tissues, including gastrointestinal tissue and peripheral blood.^59,60^ In our study, its expression was significantly higher in R compared with NR [*p*_BH_ = 0.016] prior to treatment. Previous literature suggests that olfactory receptors can communicate with gut microbiota and transduce intracellular signals, making them potential therapeutic targets in cancer.^61–63^ Additionally, chemokines or hormones that bind to G-coupled proteins can activate JAK-mediated phosphorylation of STATs, besides cytokine-mediated JAK-STAT activation.^9^ OR2L13 has previously been identified as a differentially methylated region in female Crohn’s disease patients, but no studies have associated extra-olfactory receptor signalling with IBD or response to anti-inflammatory treatment.^64^ Second, we observed hypermethylation of cg08899523, which was annotated to *FGFR2* or fibroblast growth factor receptor 2, along with a significantly lower gene expression at T1 [*p*_BH_ = 0.011] in R compared with NR. FGFR2 functions as a tyrosine kinase cell-surface receptor for fibroblast growth factors involved in cellular processes like proliferation, differentiation, migration, and apoptosis.^65^ FGFR signalling has been linked to MAPK, PI3K-Akt, and STAT-dependent signalling, making it a potential target for cancer treatment, and overexpression of *FGFR2* has been found to promote STAT1 activation in response to tofacitinib treatment.^66,67^ Previous literature also reports elevated levels of FGFR2 in peripheral blood samples of UC and Crohn’s disease patients compared with healthy controls, with increased expression of mucosal *FGFR2* in UC-related stenosis due to prolonged inflammation.^68^ Third, we observed hypermethylation of cg10518850, annotated to *LRPAP1*, in responders to tofacitinib, with a significant lower gene expression [*p*_BH_ = 0.02] at T1 compared with non-responders. LRPAP1 acts as a chaperone protein and can bind calmodulin, which can be phosphorylated by calmodulin-dependent kinase II, leading to the activation of the JAK-STAT signalling pathway.^69,70^ Furthermore, inhibition of calcium-dependent protein kinase II has been associated with down-regulation of JAK-STAT signalling in myeloid leukemia cells.^71^ LRPAP1 has been associated with mantle cell lymphoma and Alzheimer disease, but no such association with IBD or response to anti-inflammatory treatment has been reported.^72–74^ The remaining predictor CpG-associated genes [*MAGI3*,^75–78^*RPTOR*,^79–81^*PALD1*,^82,83^*SPATC1L*,^84–87^ and *ERICH1*^88–90]^, though not presenting significant differential gene expression at T1 or T2, have been associated with JAK-STAT signalling in the past.

The main strength of this study is the strict prospective selection of R and NR based on a composite endpoint consisting of centrally read endoscopic evaluation combined with clinical and/or biochemical response, ensuring high confidence in the analysed phenotype. In addition, the longitudinal design of this study allowed for exploration and selection of highly stable epigenetic biomarkers, increasing the probability of independent validation.^51^

However, there are several limitations that need to be addressed. First, the associative nature of these analyses inhibits causal inference of the observed differences in whole-blood DNA methylation and gene expression with response to tofacitinib. The fact that the majority of the identified CpG loci are located in introns, in combination with the lack of significant differences in gene expression of their associated genes, complicates the biological interpretation of our observations. Whereas all predictor CpGs, as a panel, are able to act as a good predictor for tofacitinib response, it is evident that the biological underpinnings of our observations are more complex than simply identifying inversely correlated DNA methylation and gene expression signals. Second, despite international consensus, defining endoscopic response using a decrease in endoscopic mayo score [EMS] ≥1 might misclassify patients who present an EMS of 2 upon response assessment, as these are typically not recognised as responders in clinical practice. However, out of the 16 responding patients, 12 decreased to an EMS 0 or 1. Three out of the four remaining patients presented an EMS of 2 in only a single segment, all other segments being either EMS 0 or 1. However, these patients additionally presented a drop of ≥2 points or 50% in UCEIS, combined with both clinical and biochemical response. Only a single patient went from an EMS 3 pancolitis to an EMS 2 pancolitis yet also presented ≥50% decrease in total UCEIS, SCCAI, and faecal calprotectin. We therefore were confident in our selection of these patients as responders. Third, the observed differences in PB DNA methylation and the predictive model have been generated in an explorative discovery cohort, with internal validation using a 20% unseen proportion of that dataset. Although using several techniques to mitigate overfitting [ie, 10-fold cross-validation, stability selection, and pairwise imputation], the lack of a true external cohort might have resulted in an overestimated AUC performance. Additional analyses in a similar, collected, external cohort with a larger sample size are necessary to further validate our findings for clinical practice. Future research could therefore use our explorative observations as a reference when estimating the necessary samples size. Fourth, our study did not include a different comparator mode of action nor disease type [ie, psoriasis or rheumatoid arthritis]. We are therefore unable to claim drug or disease specificity of our response-associated markers.

In conclusion, we report on a novel panel of 53 hyperstable epigenetic markers that are associated with clinical, biochemical, and endoscopic response to tofacitinib treatment in UC. Our data provide targets that need to be further investigated in order to better understand the mechanism underlying tofacitinib response. Whereas independent validation of our finding is necessary, this study serves as an initial step towards more personalised treatment of UC patients.

## Supplementary Data

Supplementary data are available at *ECCO-JCC* online.

jjad129_suppl_Supplementary_Figure_S1

jjad129_suppl_Supplementary_Figure_S2

jjad129_suppl_Supplementary_Figure_S3

jjad129_suppl_Supplementary_Figure_S4

jjad129_suppl_Supplementary_Tables

jjad129_suppl_Supplementary_Data
