# Supplementary figures and images for: Peripheral Blood DNA Methylation Signatures and Response to Tofacitinib in Moderate-to-severe Ulcerative Colitis
Source: J Crohns Colitis. 2023 Aug 1;18(8):1179–89. doi: 10.1093/ecco-jcc/jjad129 (PMC11324342; doi:10.1093/ecco-jcc/jjad129)

# ROC

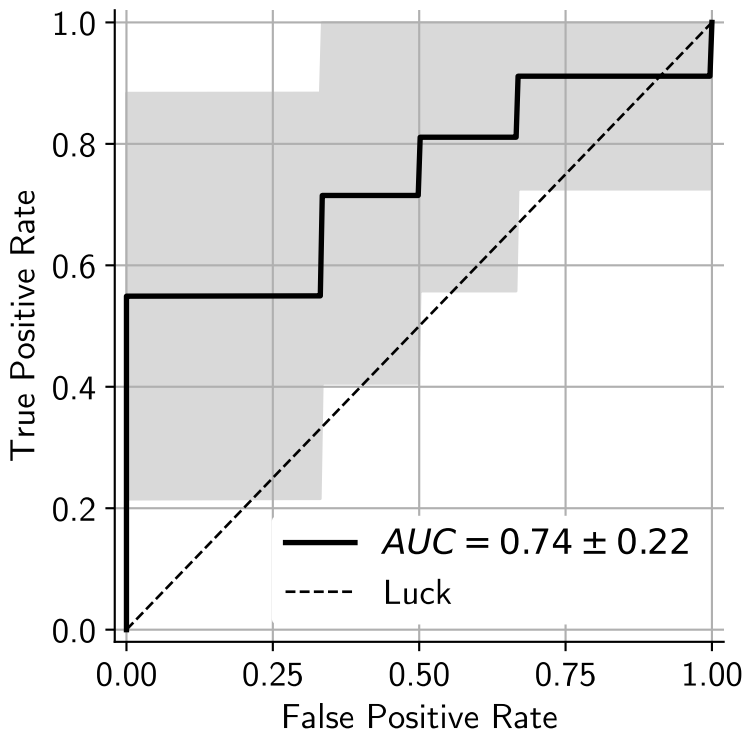

Supplement: jjad129_suppl_Supplementary_Figure_S1 [file jjad129_suppl_supplementary_figure_s1.pdf]

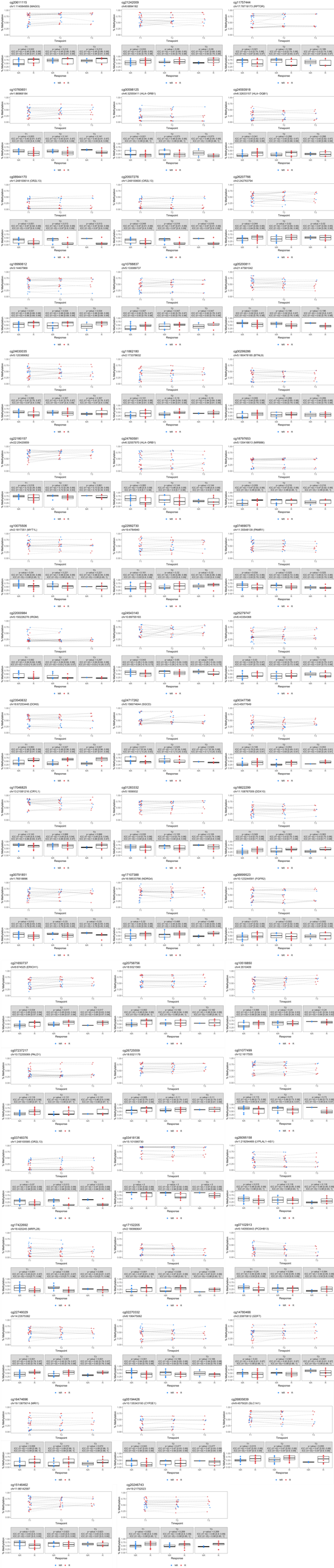

Supplement: jjad129_suppl_Supplementary_Figure_S2 [file jjad129_suppl_supplementary_figure_s2.pdf]

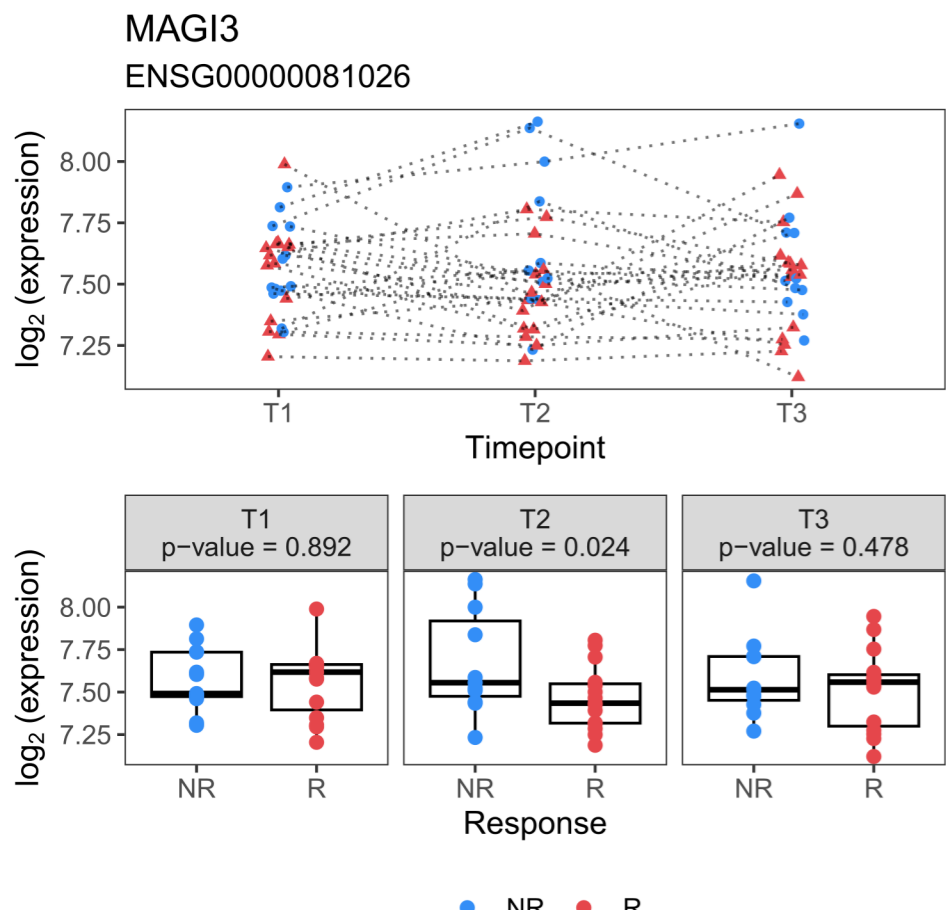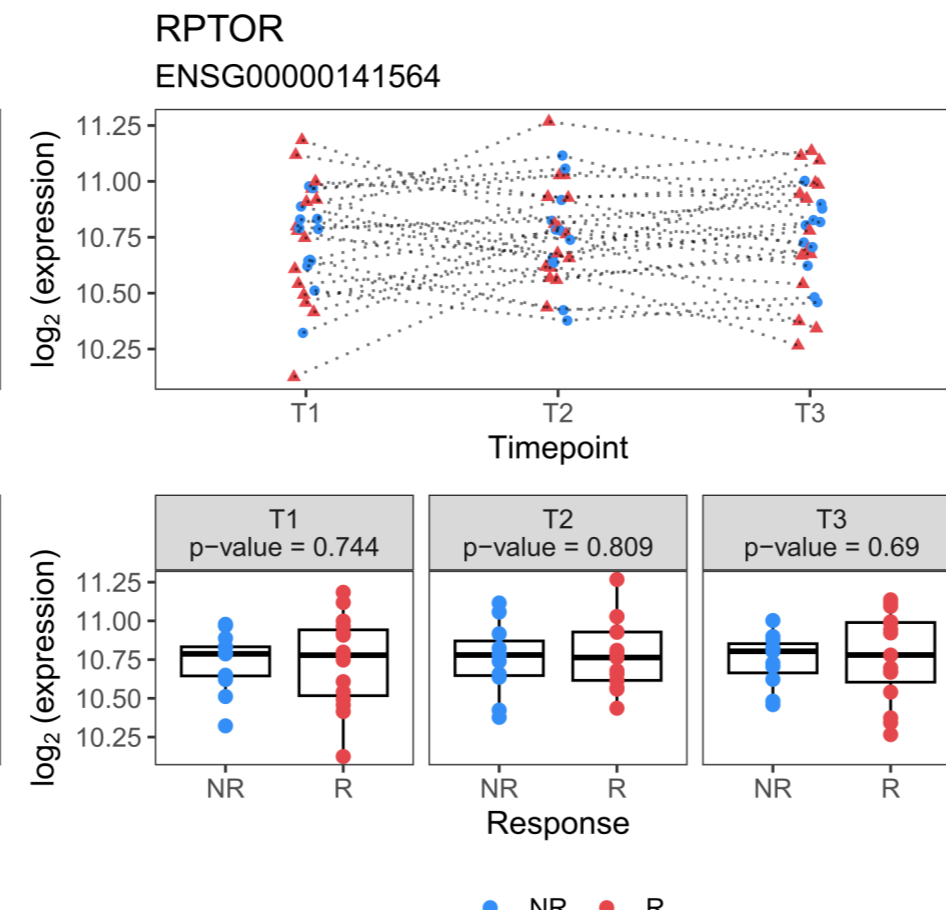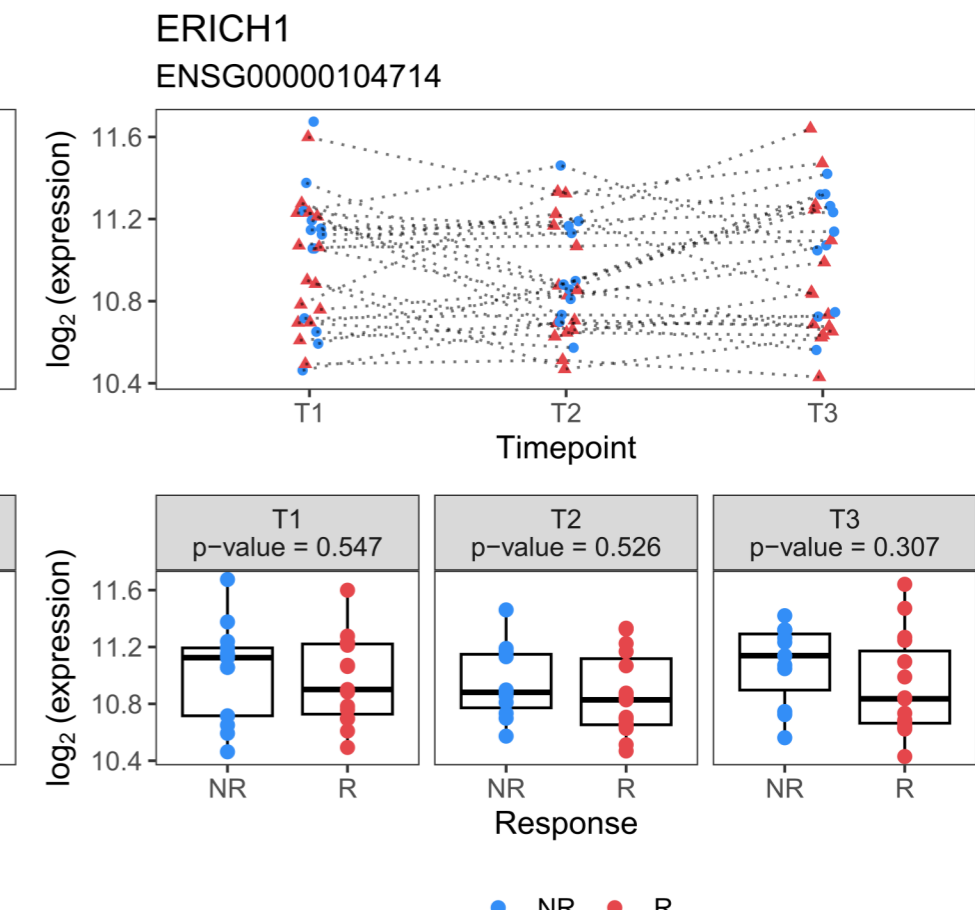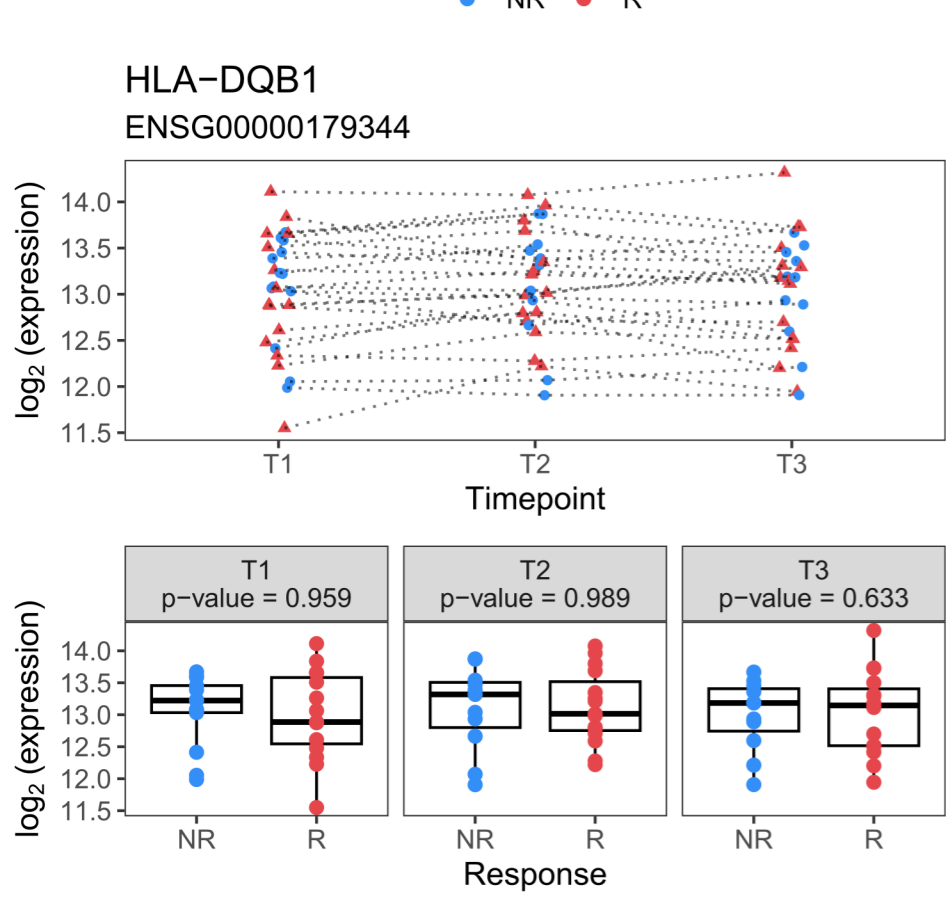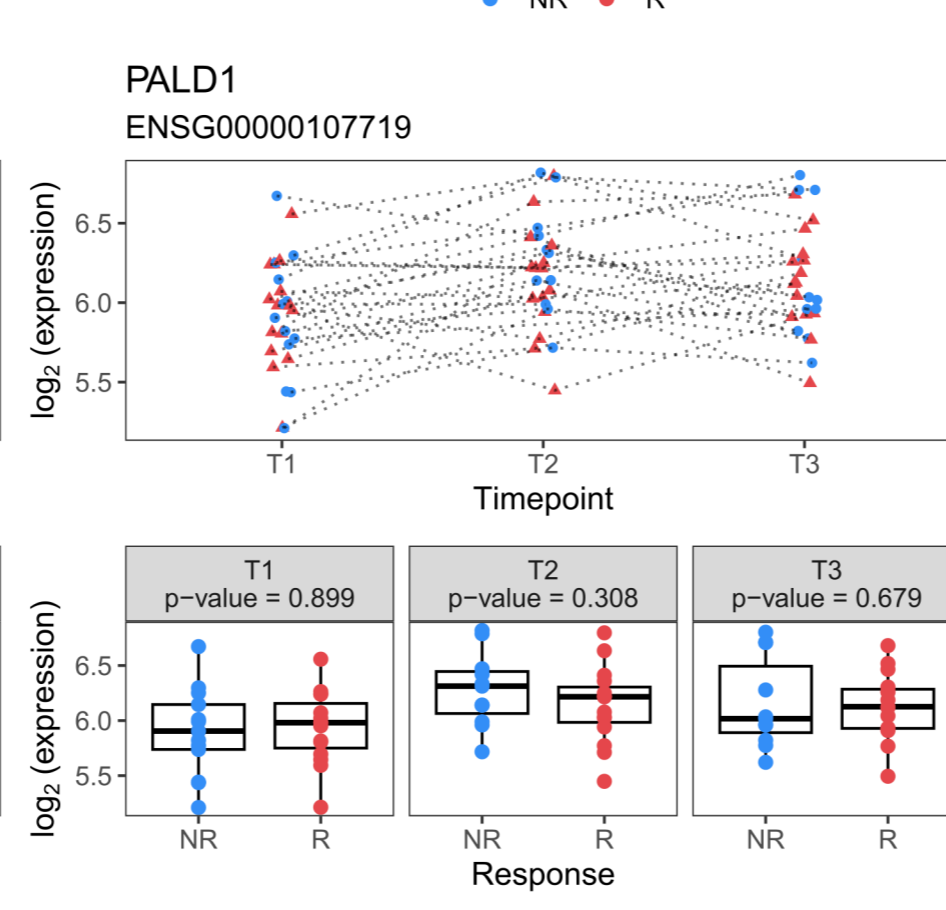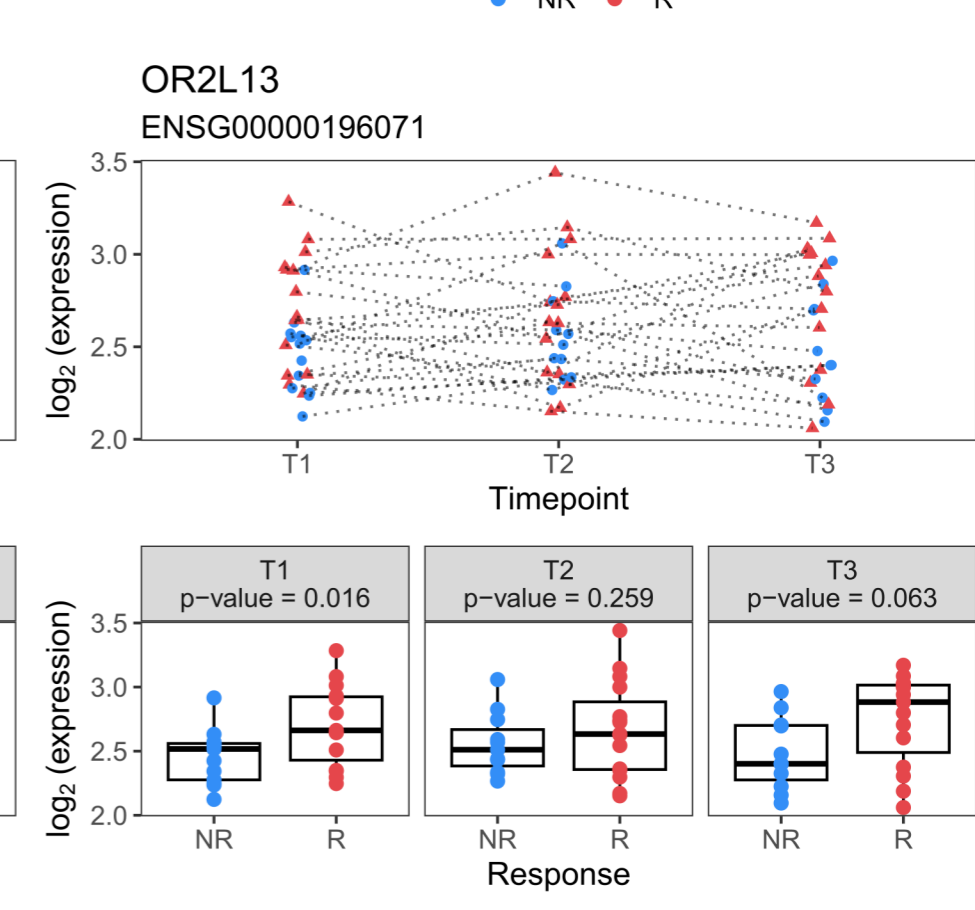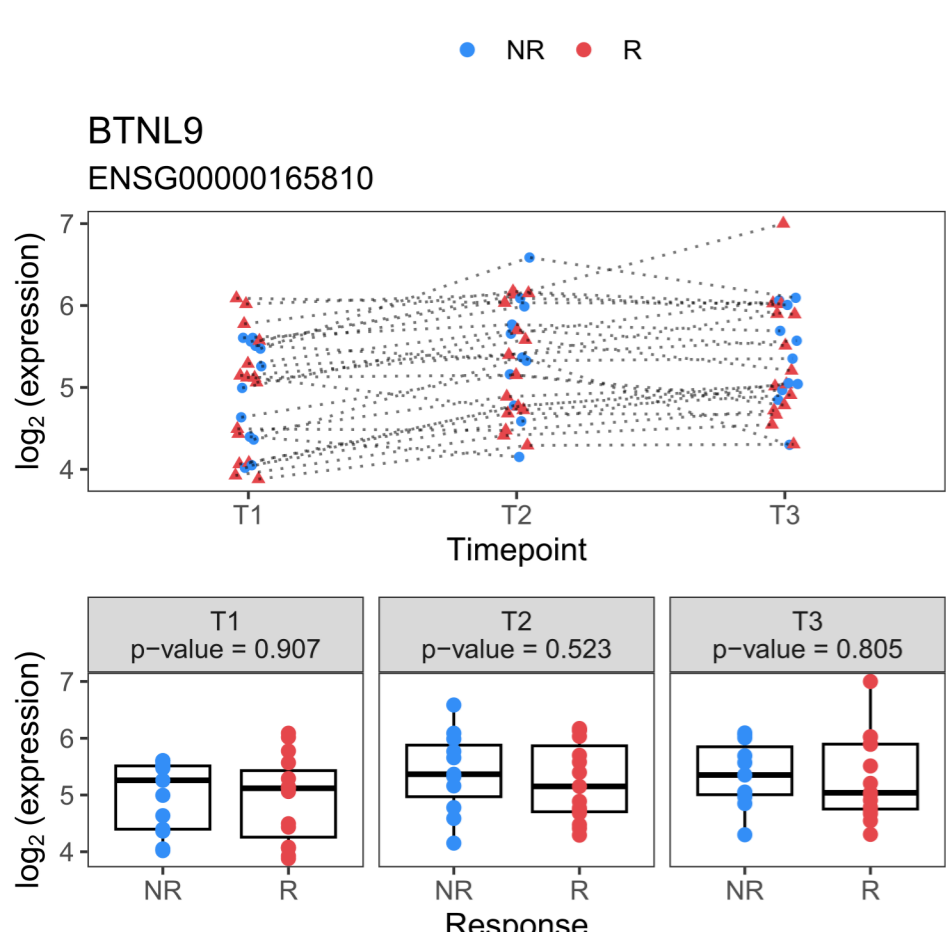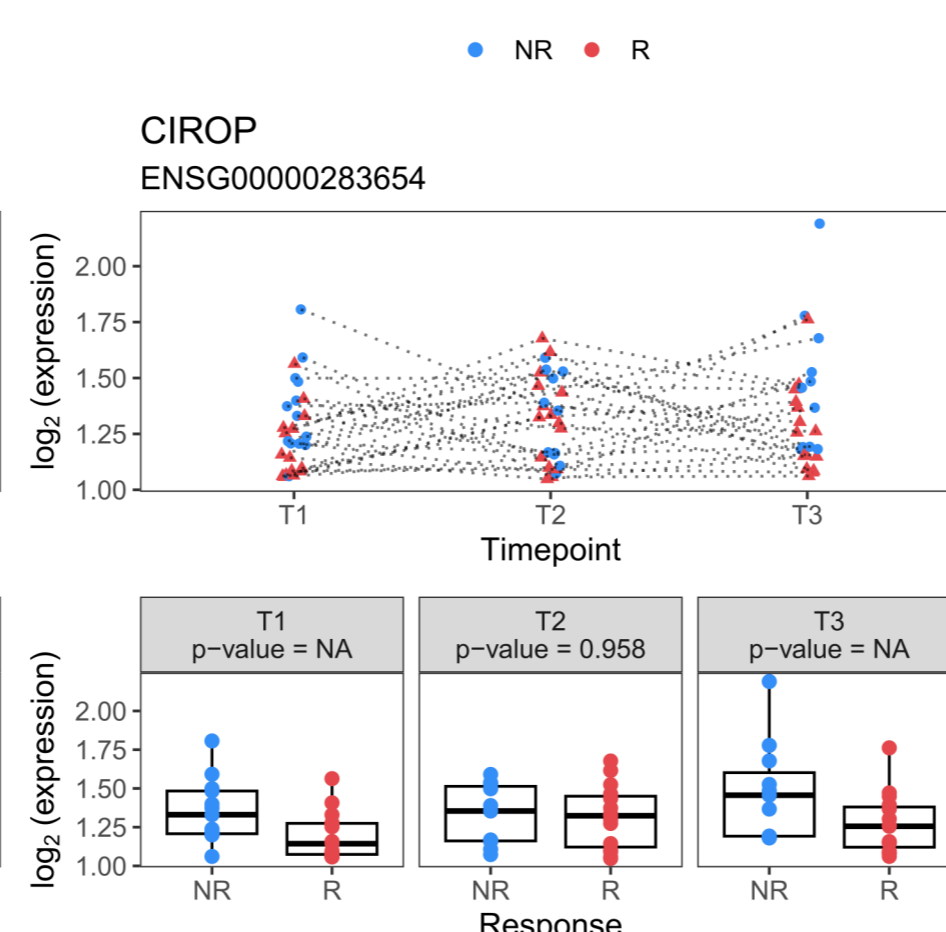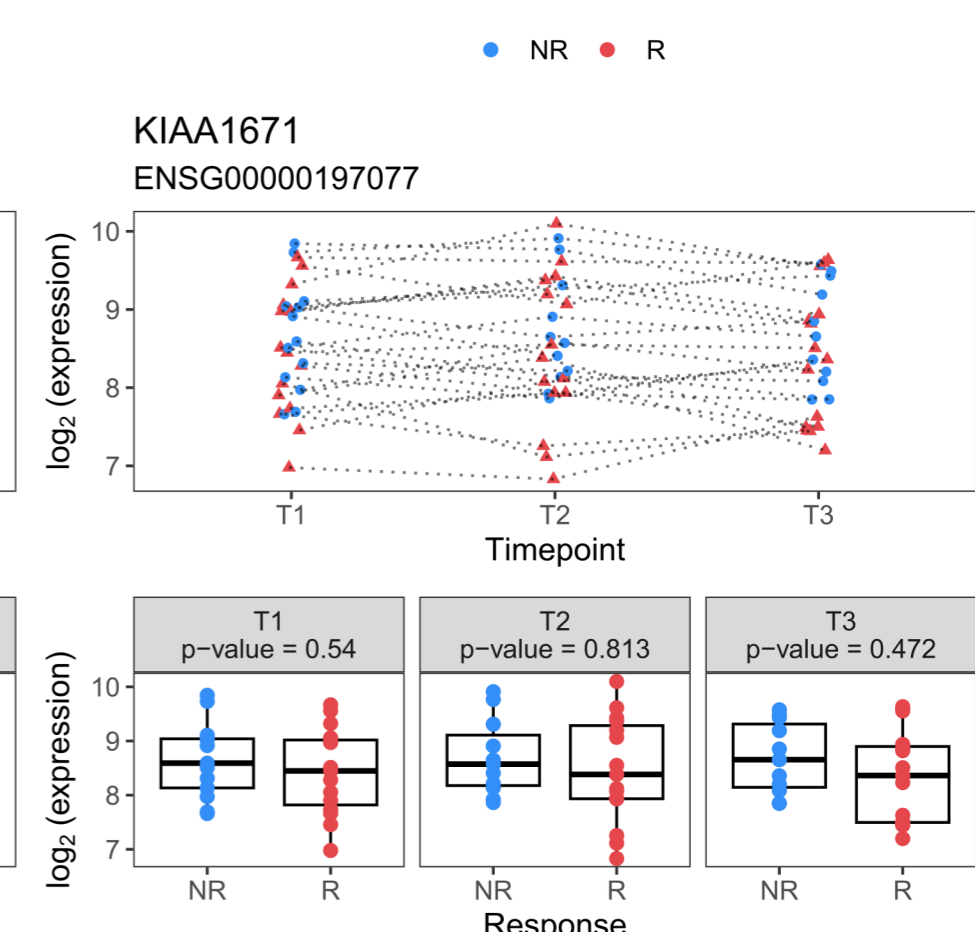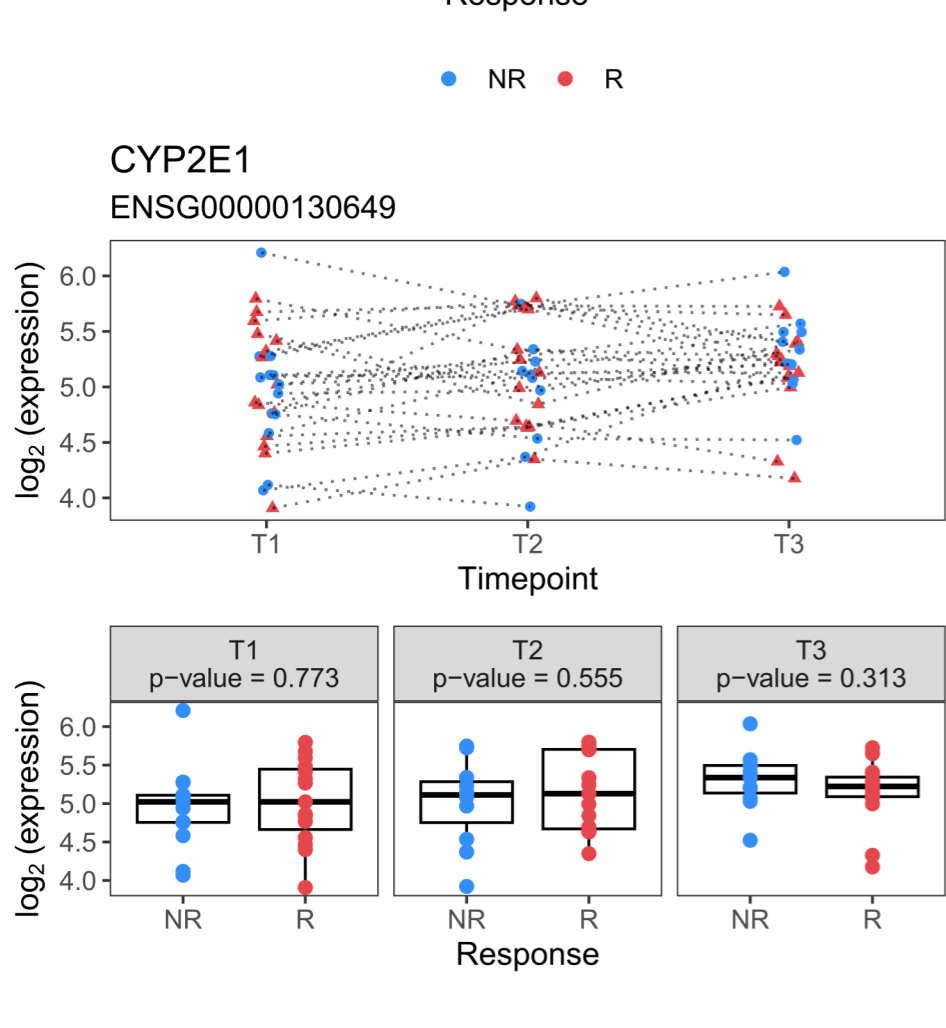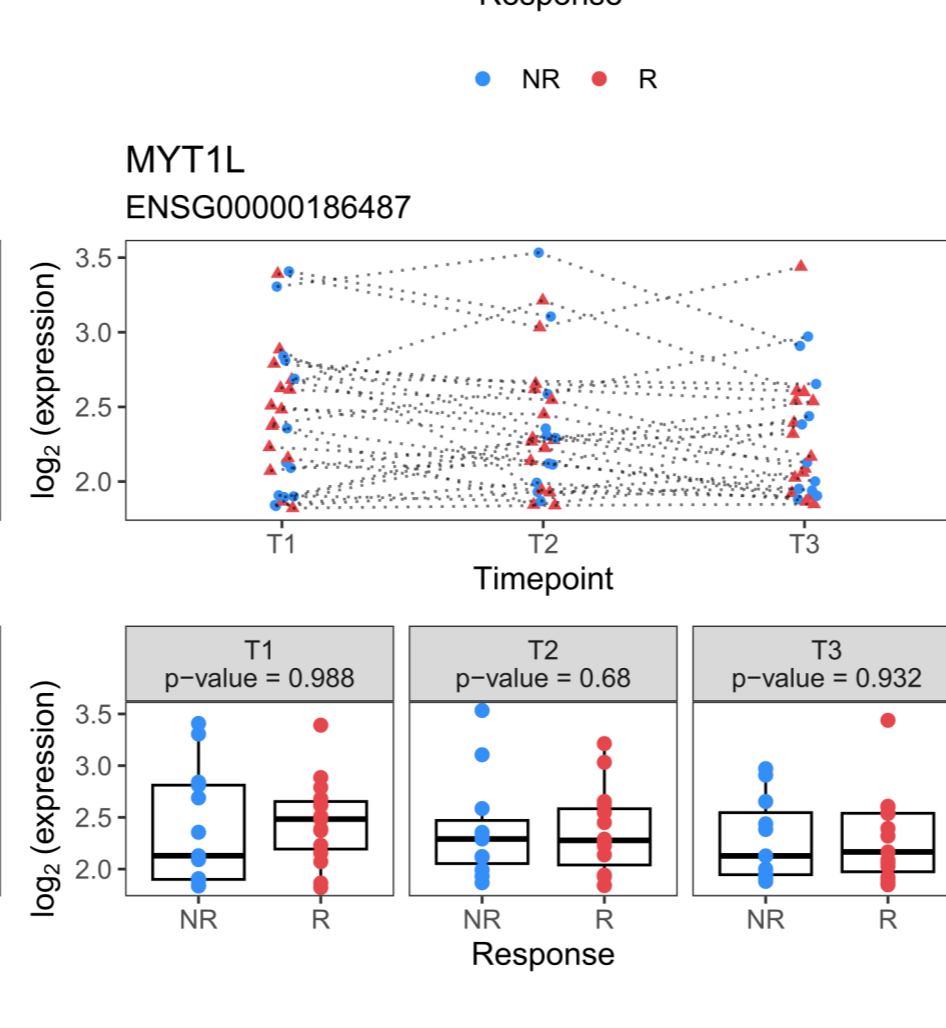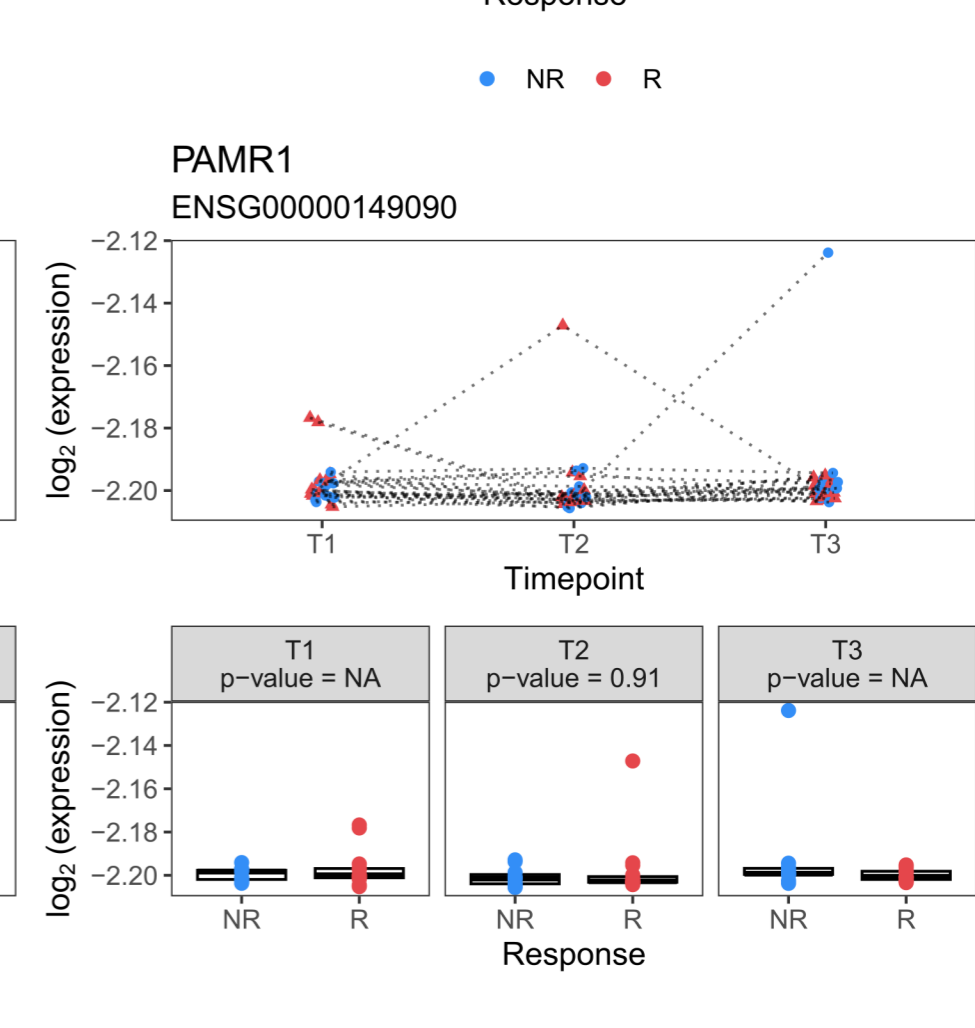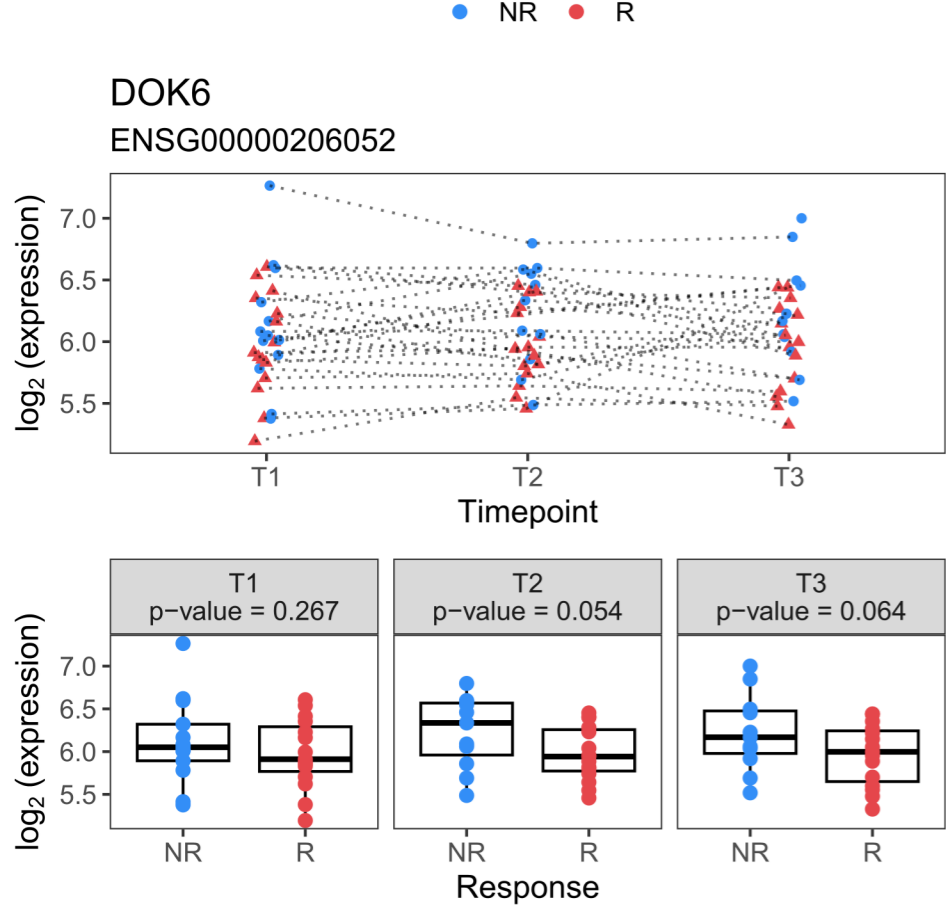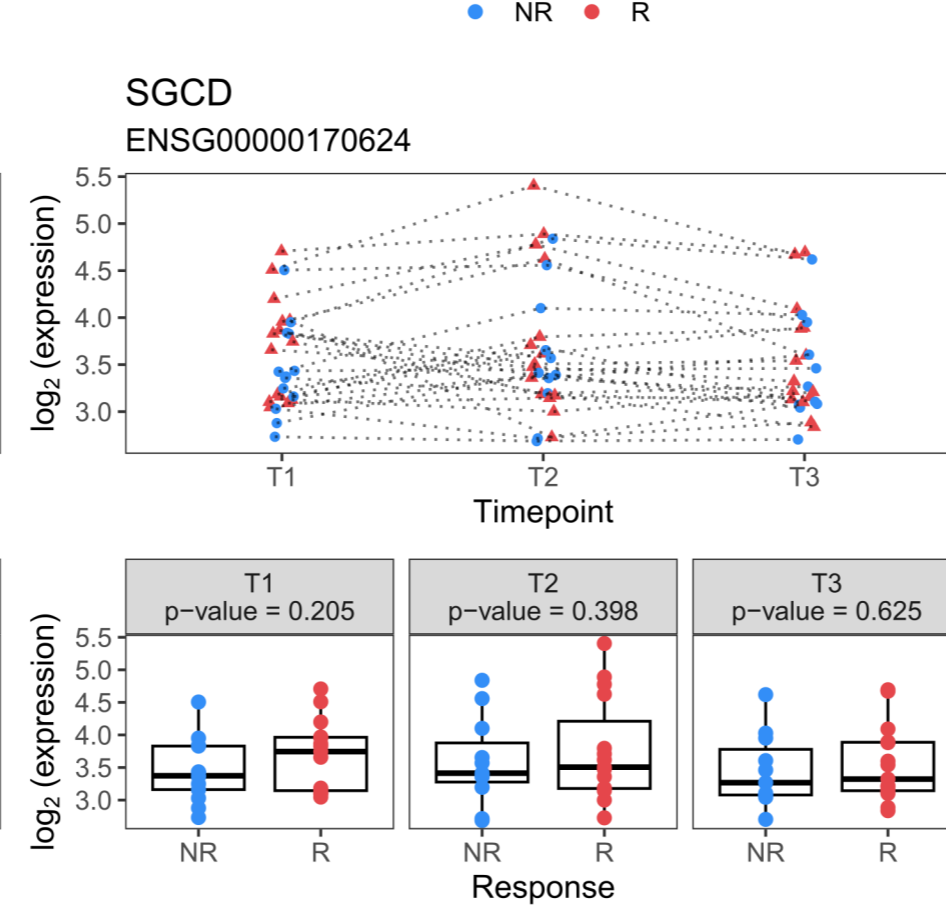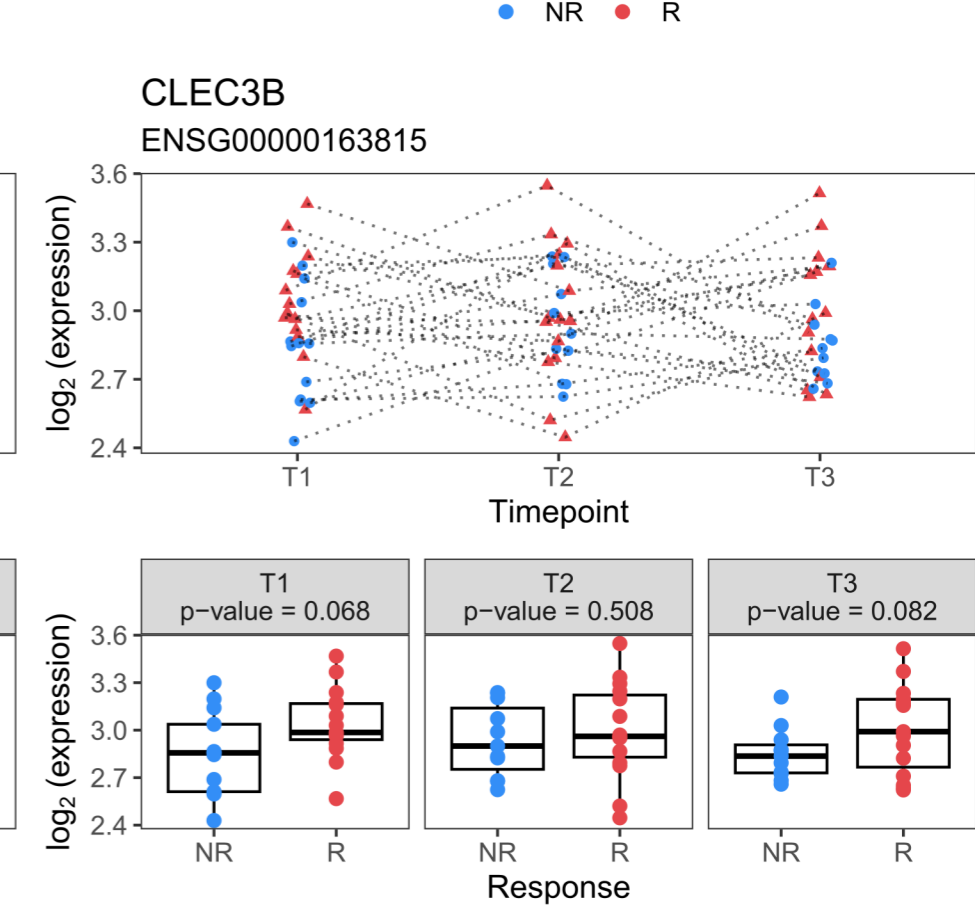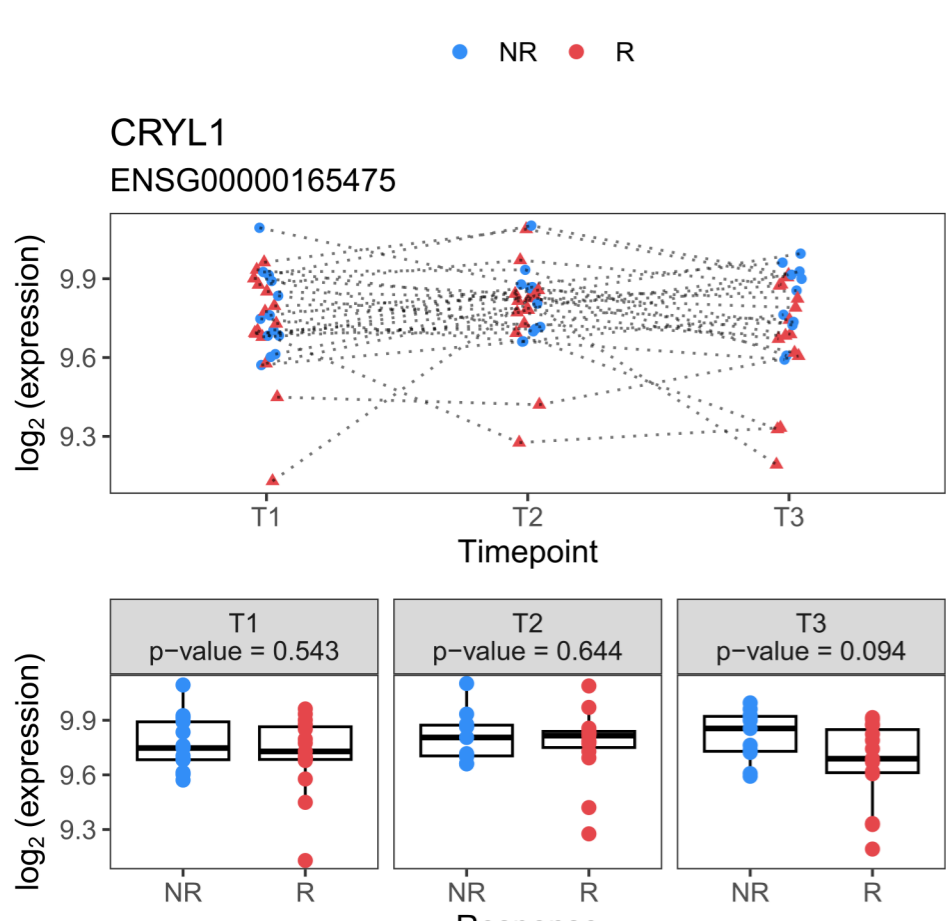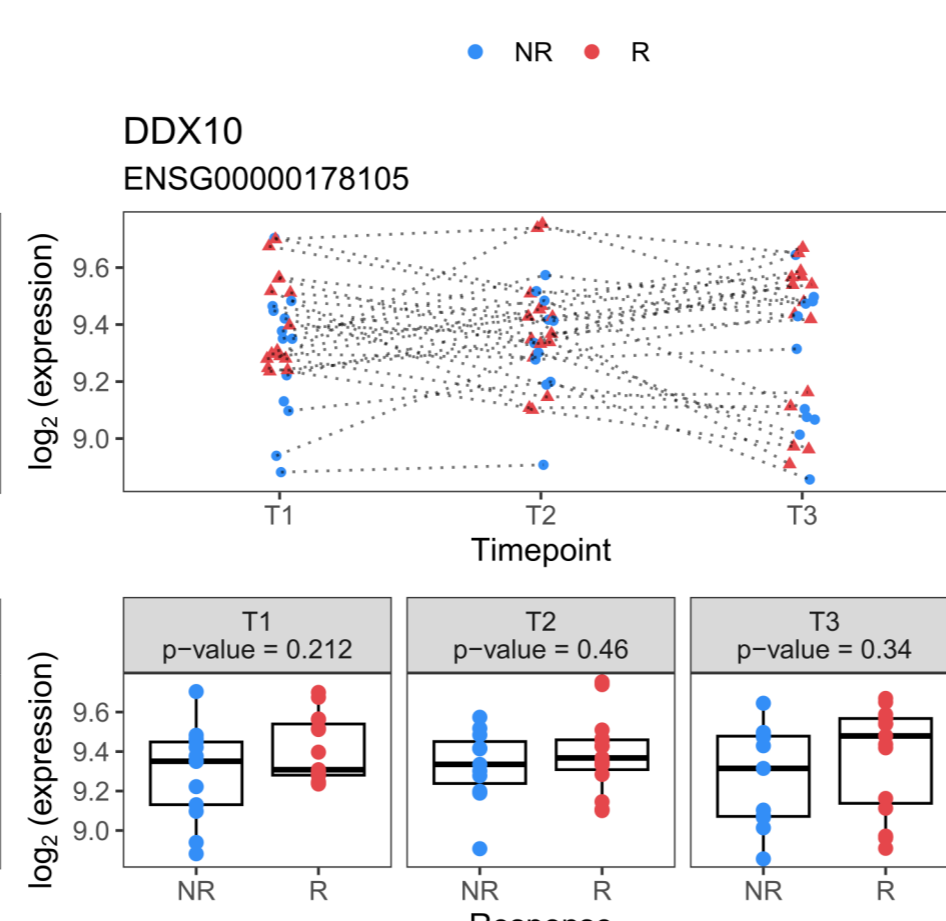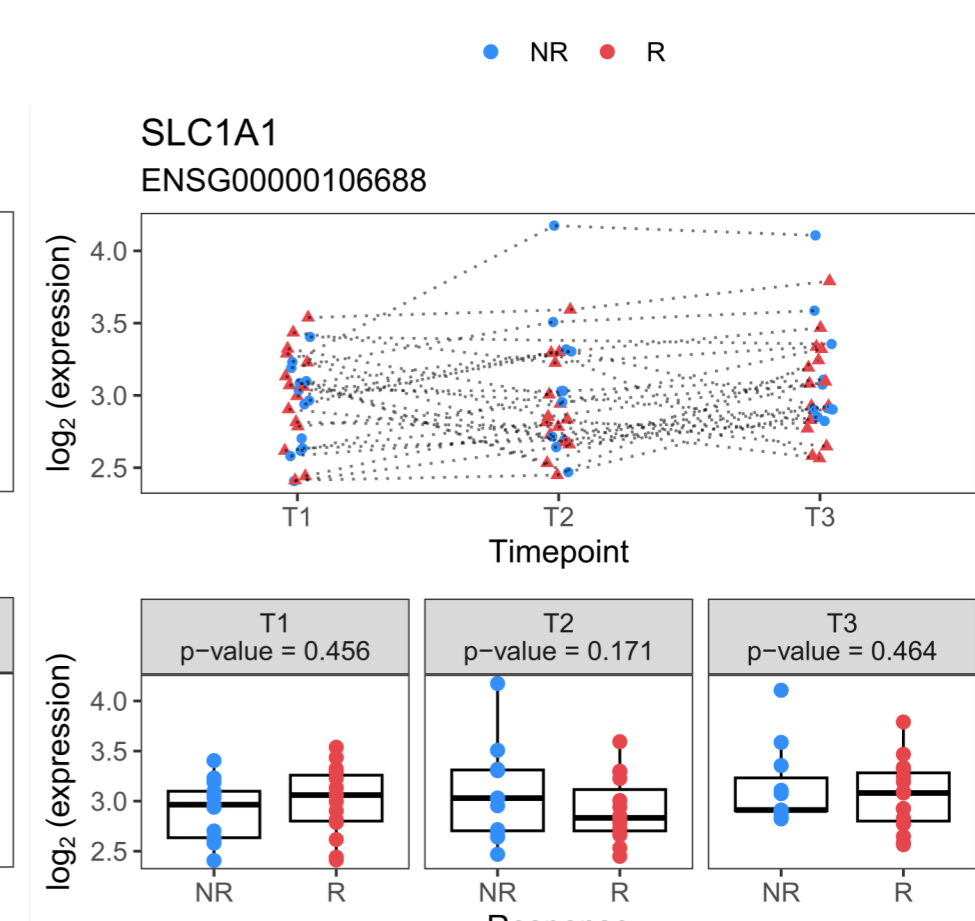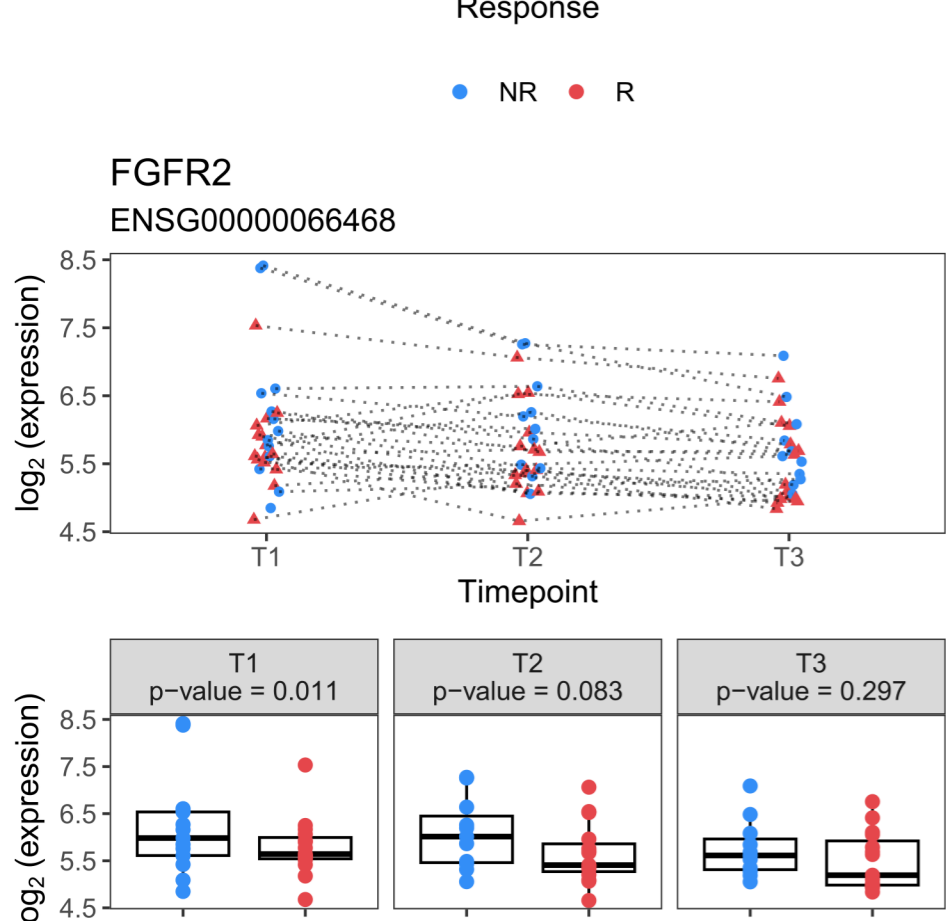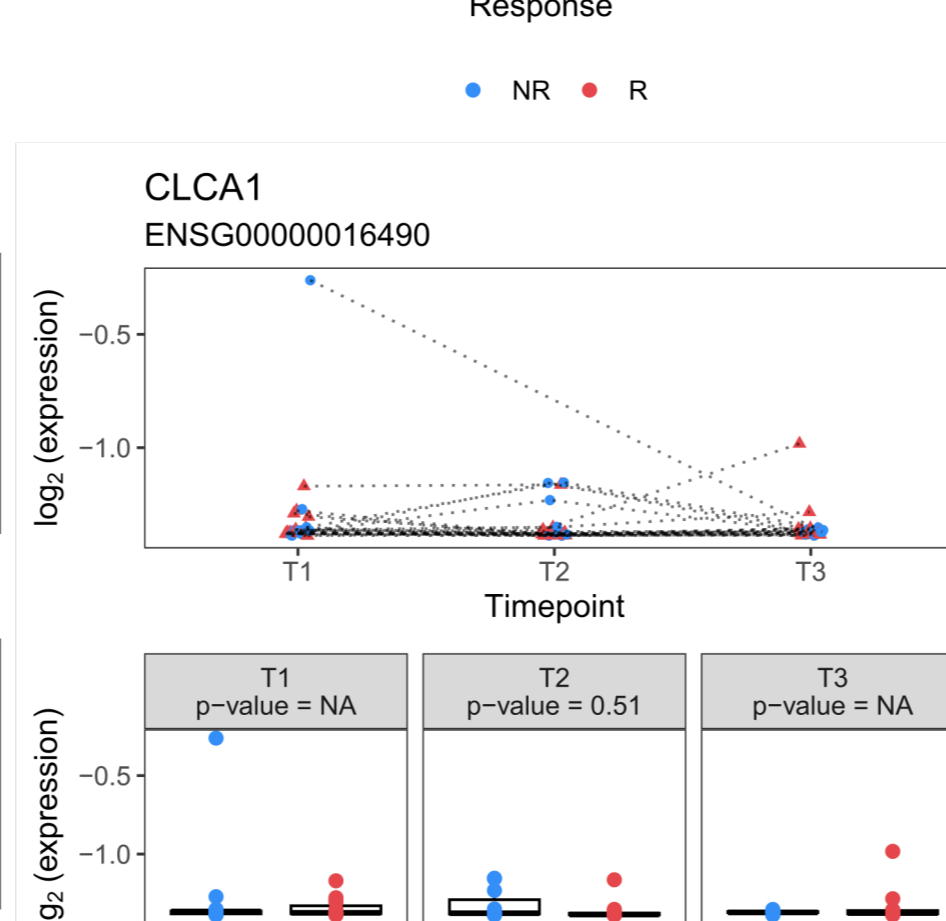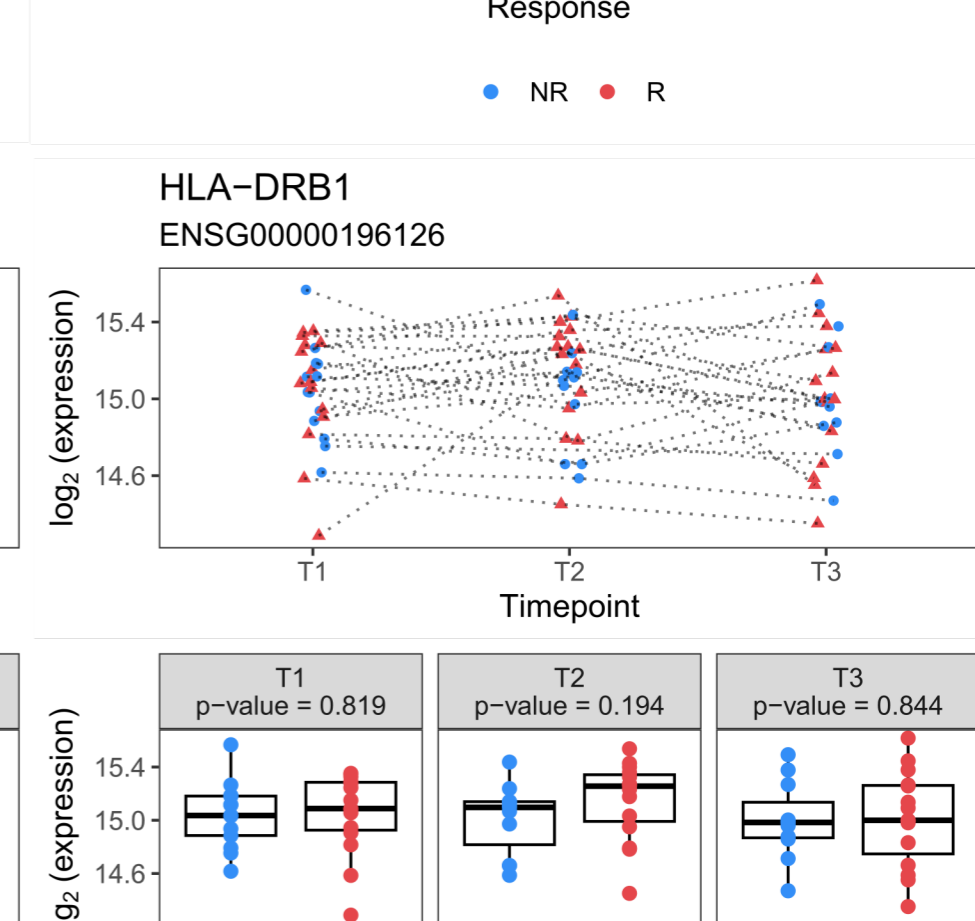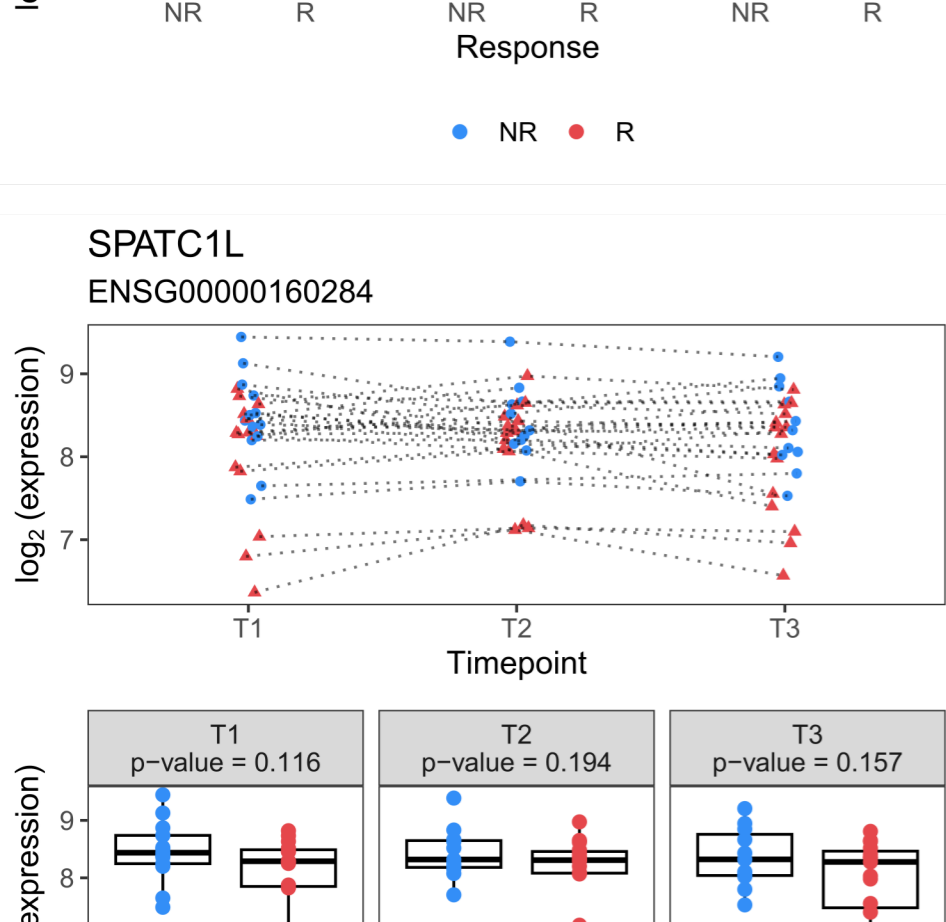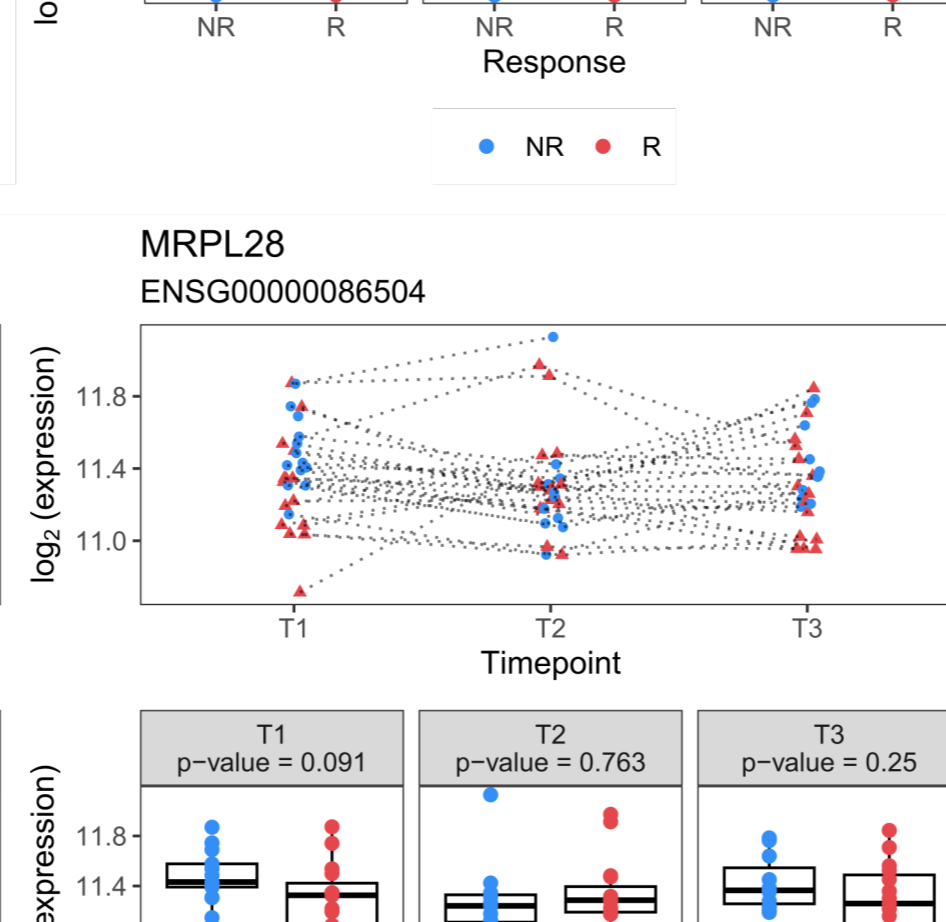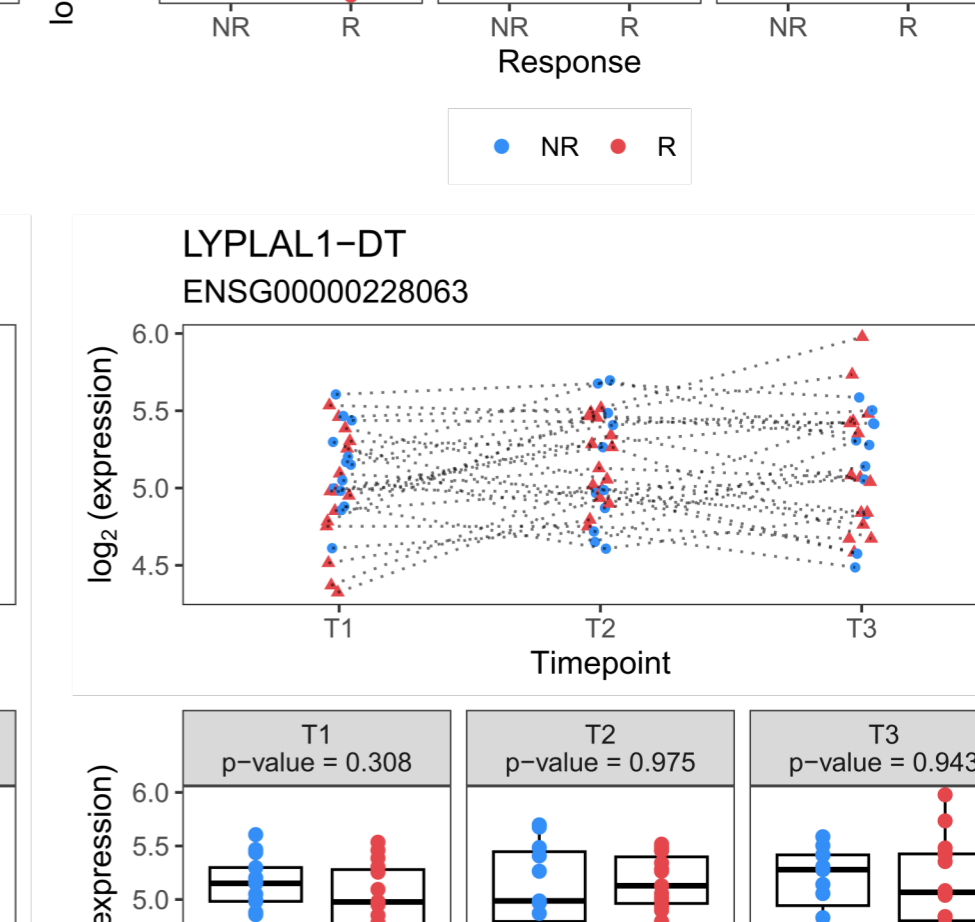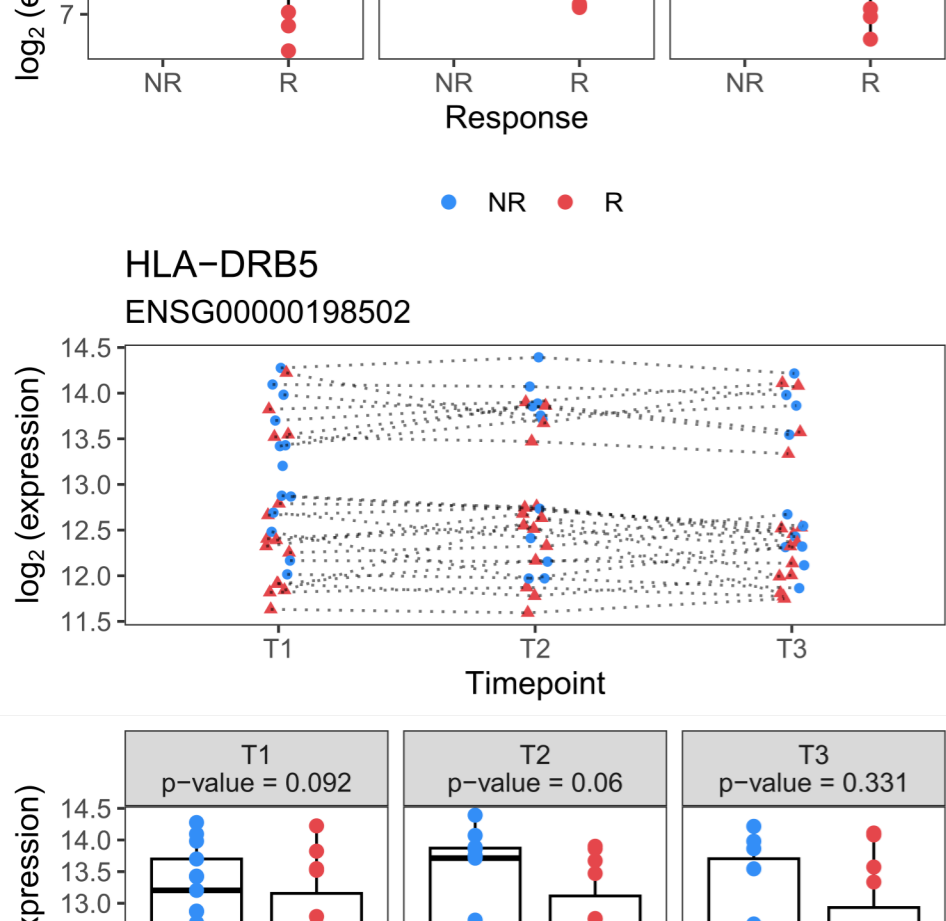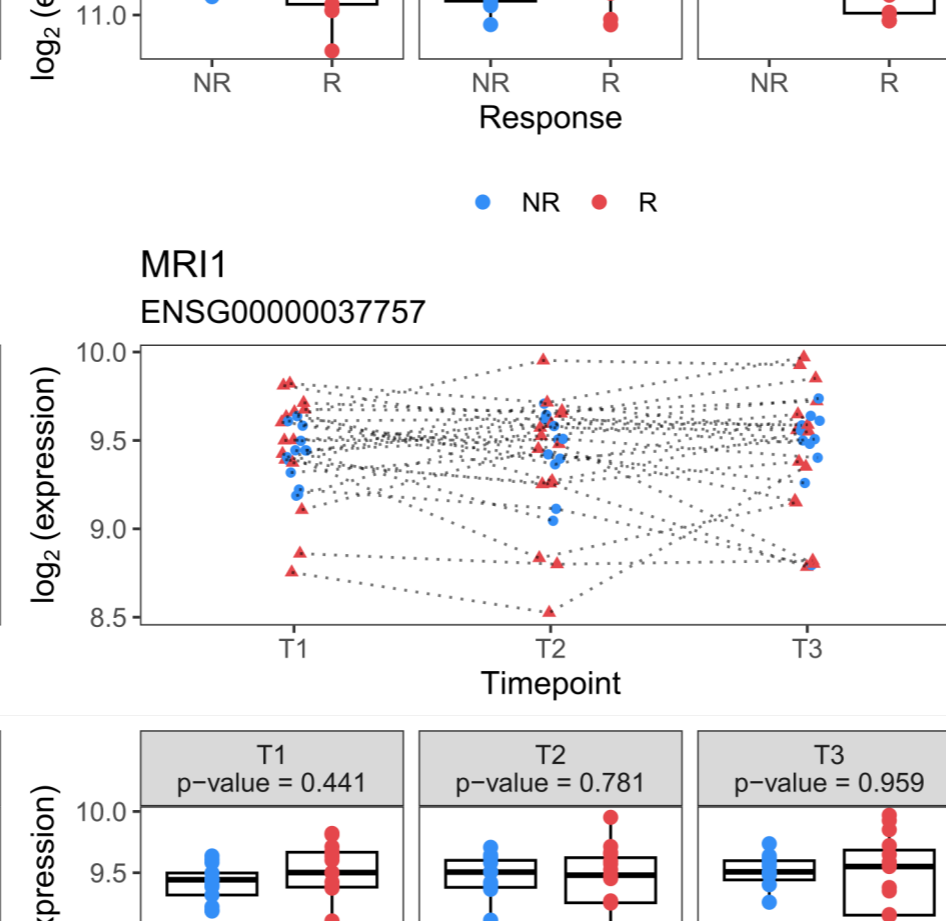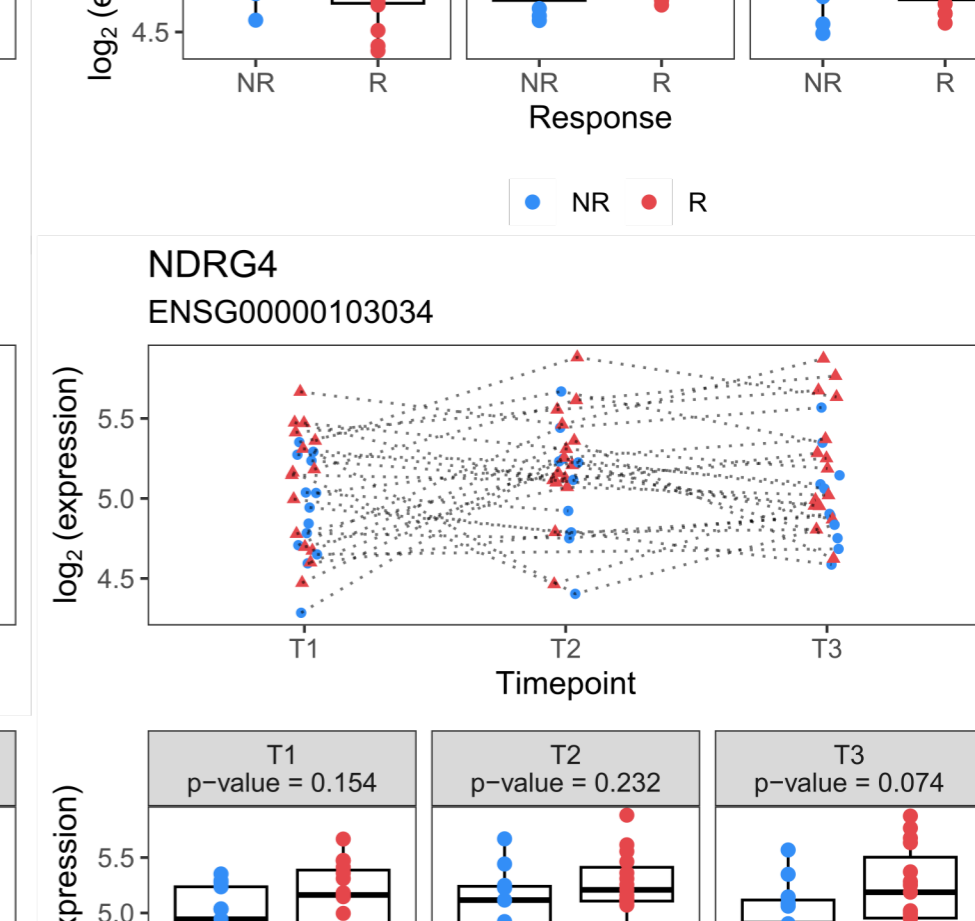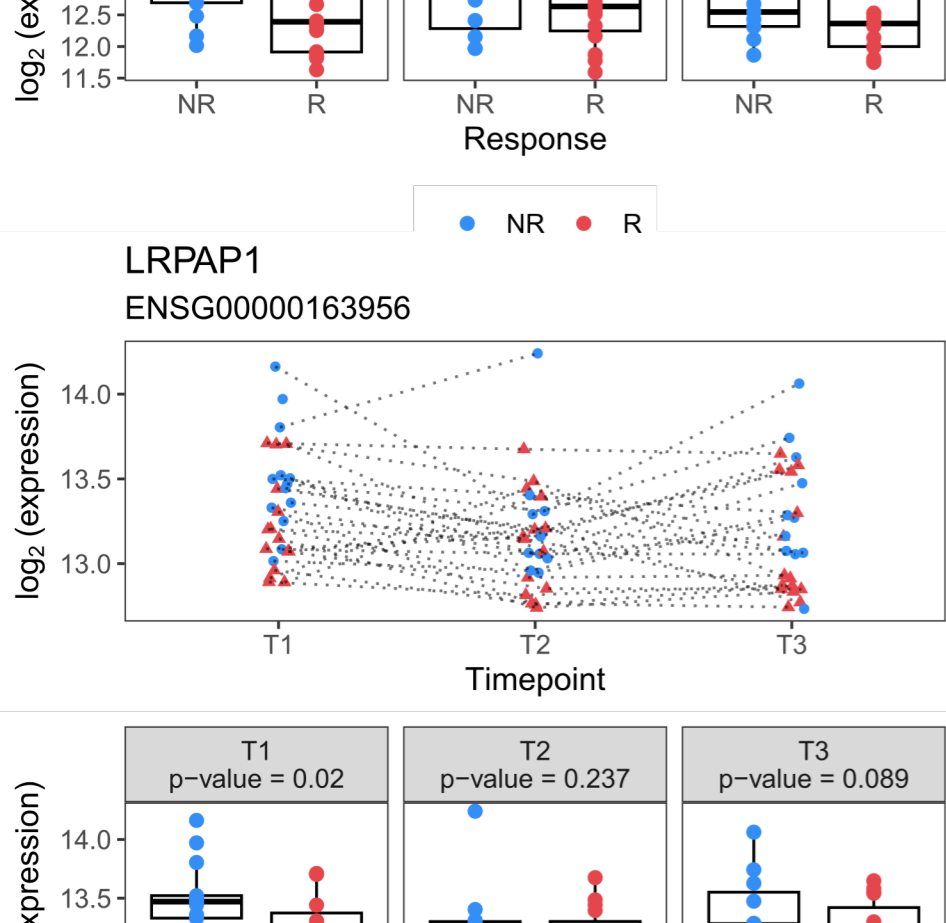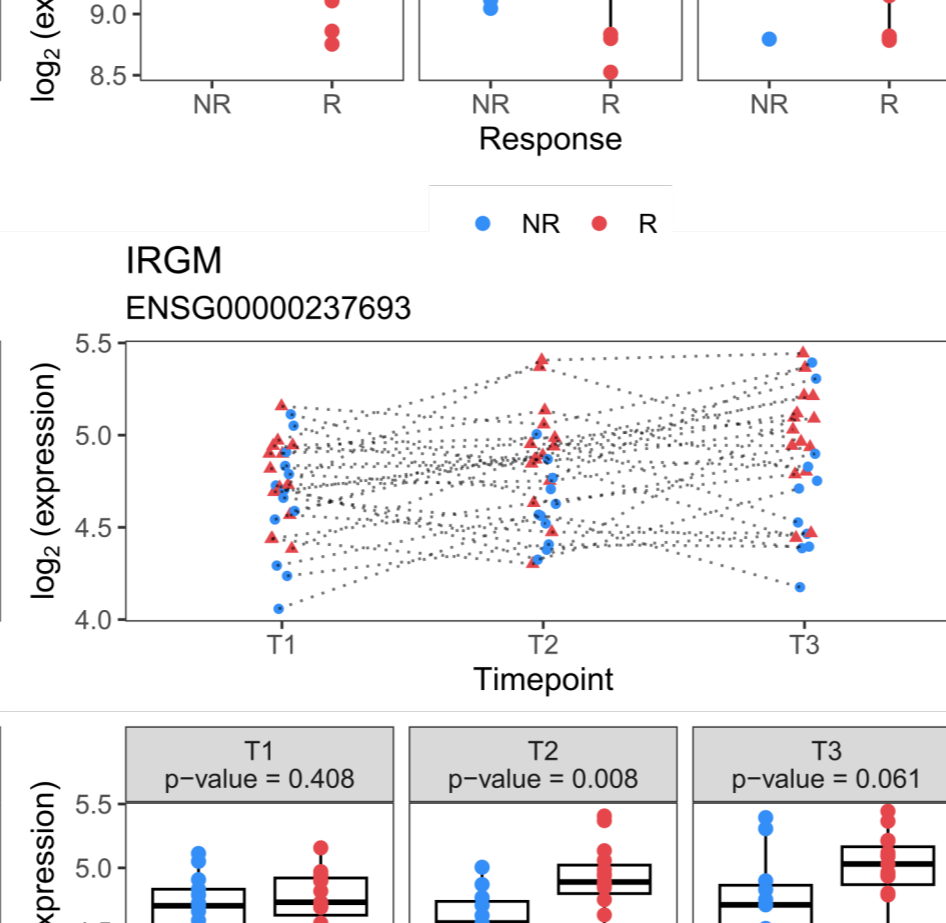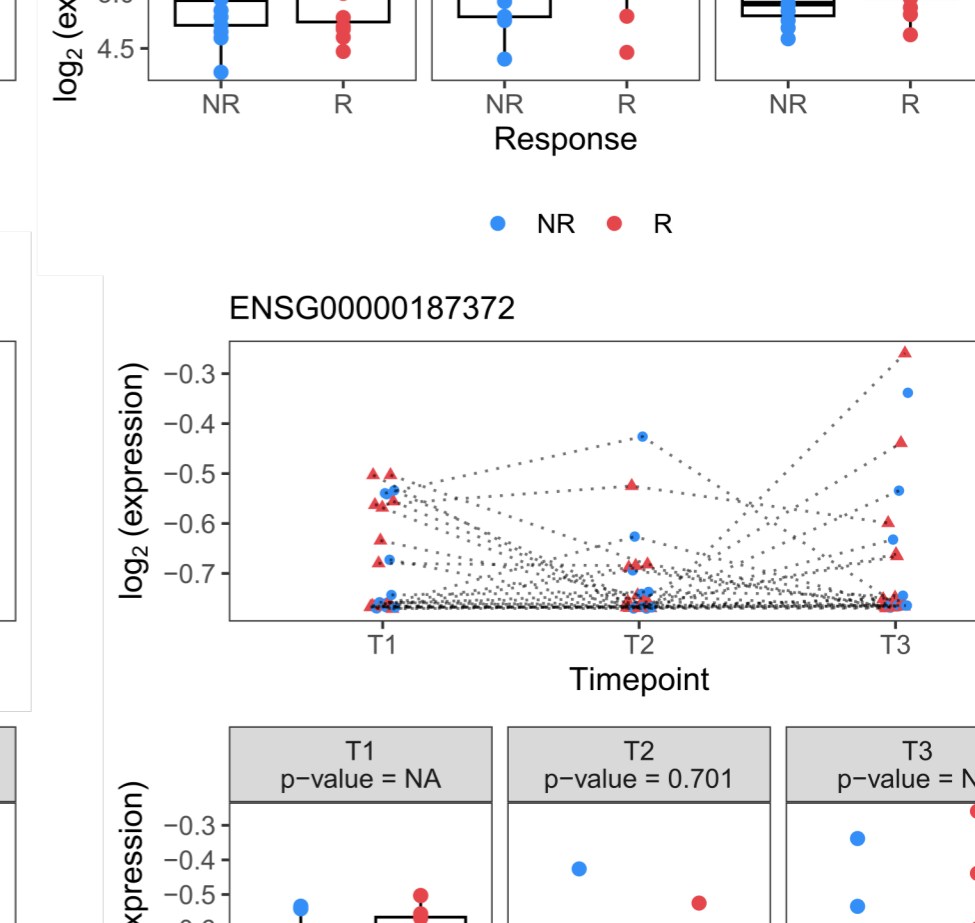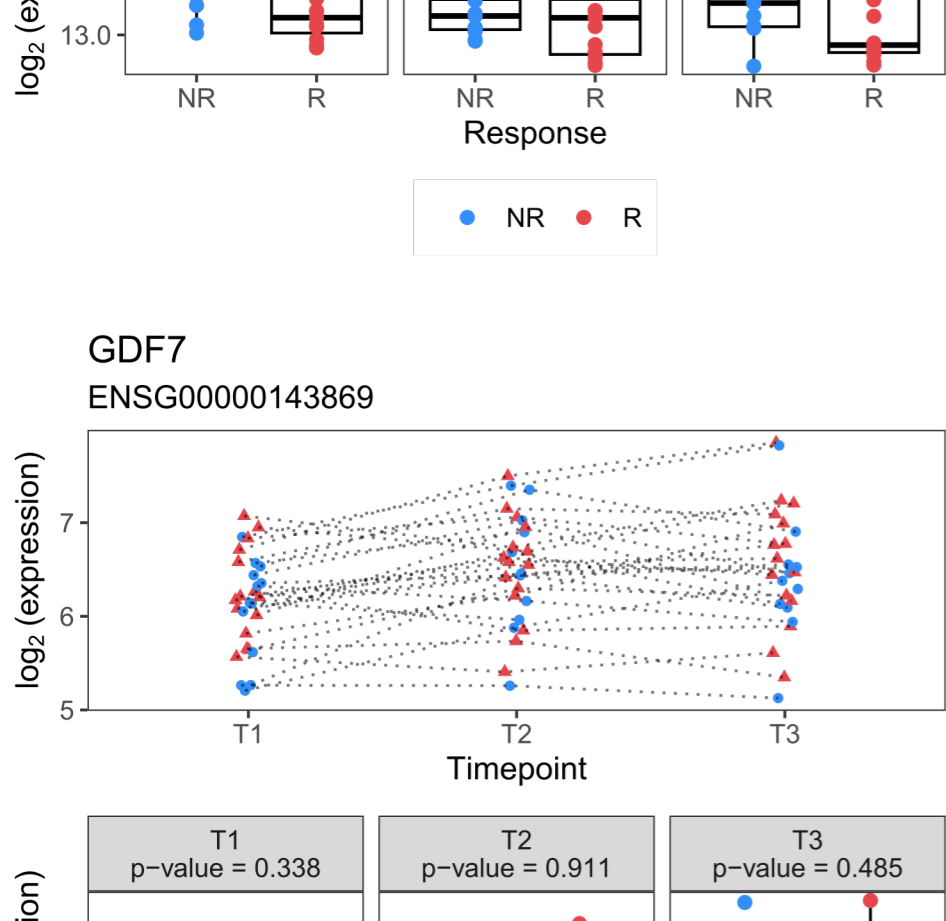

Supplement: jjad129_suppl_Supplementary_Figure_S3 [file jjad129_suppl_supplementary_figure_s3.pdf]

Temporal stability predictor CpGs

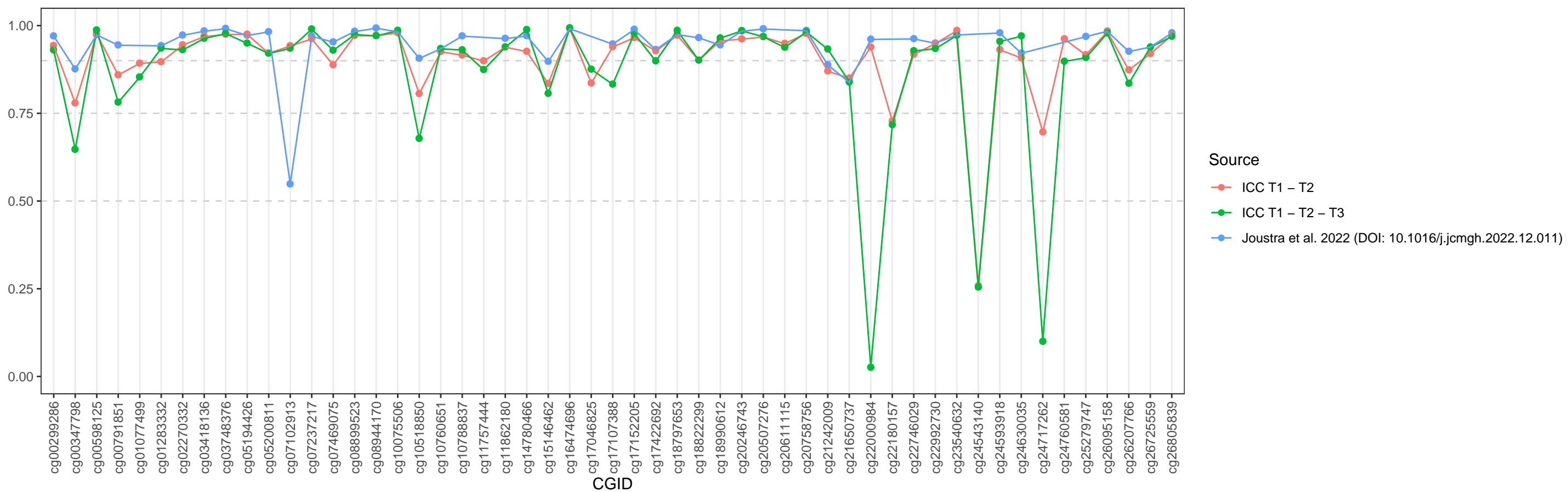

Supplement: jjad129_suppl_Supplementary_Figure_S4 [file jjad129_suppl_supplementary_figure_s4.pdf]
