# Supplementary material for: Peripheral Blood DNA Methylation Signatures and Response to Tofacitinib in Moderate-to-severe Ulcerative Colitis
Source: J Crohns Colitis. 2023 Aug 1;18(8):1179–89. doi: 10.1093/ecco-jcc/jjad129 (PMC11324342; doi:10.1093/ecco-jcc/jjad129)
Supplement: jjad129_suppl_Supplementary_Data [file jjad129_suppl_supplementary_data.docx]

# **Supplementary figure legends**

**Figure S1** receiver operating characteristics plot showing mean AUC performance upon internal validation of the predictor CpGs with long-term (week 104) response outcome.

**Figure S2** Boxplots of the DNA methylation for all predictor CpGs.

Visualization of the DNA methylation for each individual sample before (T1) and at 8 weeks into tofacitinib treatment (T2) time colored by response. Red represents responders and blue represents non-responders. The top plot represents a scatterplot of the patient over time, with dashed lines connecting samples obtained from the same patient. The bottom plots represent boxplots for T1 and T2 separately, comparing responders and non-responders annotated with the differential methylation p-value.

**Figure S3** Boxplots and gene expression for genes associated with the predictor CpGs.

Visualization of the gene expression for each individual sample before (T1) and at 8 weeks into tofacitinib treatment (T2) time colored by response. Red represents responders and blue represents non-responders. The top plot represents a scatterplot of the patient over time, with dashed lines connecting samples obtained from the same patient. The bottom plots represent boxplots for T1 and T2 separately, comparing responders and non-responders annotated with the differential expression p-value.

**Figure S4** Temporal stability of the predictor CpGs over time

Visualization of the intra-class correlation coefficients of the predictor CpG loci as calculated by compared T1 vs. T2 (pink), T1 vs. T2 vs. T3 (green) and using the long-term term stability dataset published by Joustra et al. (blue). As observed, the majority of the predictor CpG loci present stable methylation profiles in all three separate analyses.
